# CD74 as a prognostic and M1 macrophage infiltration marker in a comprehensive pan-cancer analysis

**DOI:** 10.1038/s41598-024-58899-7

**Published:** 2024-04-07

**Authors:** Ruo Qi Li, Lei Yan, Ling Zhang, Yanli Zhao, Jing Lian

**Affiliations:** 1 Department of Pathology, Cancer Hospital Affiliated to Shanxi Province Cancer Hospital/Shanxi Hospital Affiliated to Cancer Hospital, Chinese Academy of Medical Sciences/Cancer Hospital Affiliated to Shanxi Medical University, Taiyuan, China; 2 General Surgery Department, Third Hospital of Shanxi Medical University, Shanxi Bethune Hospital, Shanxi Academy of Medical Sciences, Tongji Shanxi Hospital, Taiyuan, 030032 China; 3 Department of Orthopedics, The Second Hospital of Shanxi Medical University, Shanxi Key Laboratory of Bone and Soft Tissue Injury Repair, 382 Wuyi Road, Taiyuan, Shanxi China; 4 Department of Pathology, Shanxi Medical University, Taiyuan, 030001 Shanxi China

**Keywords:** Pan-cancer, Prognosis, Immune infiltration, M1 macrophage, Molecular docking, Tumour biomarkers, Data processing

## Abstract

CD74 is a type-II transmembrane glycoprotein that has been linked to tumorigenesis. However, this association was based only on phenotypic studies, and, to date, no in-depth mechanistic studies have been conducted. In this study, combined with a multi-omics study, CD74 levels were significantly upregulated in most cancers relative to normal tissues and were found to be predictive of prognosis. Elevated CD74 expression was associated with reduced levels of mismatch-repair genes and homologous repair gene signatures in over 10 tumor types. Multiple fluorescence staining and bulk, spatial, single-cell transcriptional analyses indicated its potential as a marker for M1 macrophage infiltration in pan-cancer. In addition, CD74 expression was higher in BRCA patients responsive to conventional chemotherapy and was able to predict the prognosis of these patients. Potential CD74-activating drugs (HNHA and BRD-K55186349) were identified through molecular docking to CD74. The findings indicate activation of CD74 may have potential in tumor immunotherapy.

## Introduction

Cancer contributes significantly to worldwide mortality^1^ and is also a major burden to public health. According to the Global Cancer Research Center (GLOBOCAN), in 2020 there were 19.3 million new cancer cases (18.1 million, excluding non-melanoma skin cancers) and nearly 10 million cancer deaths (9.9 million, excluding non-melanoma skin cancers) worldwide. The global cancer burden is projected to reach 28.4 million cases by 2040, a 47% increase from 2020^1^. Despite reductions in mortality resulting from surgery and early screening, the characteristic heterogeneity of tumors, together with their tendency to recur and metastasize, contribute to the overall poor prognosis and survival rates of many cancers^2^. The use of prognosis-related biomarkers and specific patient characteristics are both key to treatment and improvement in outcomes^3^. Despite the growing acceptance of personalized cancer treatments, there remains a gap between the discovery and clinical use of prognostic biomarkers^4^. Healthcare providers may feel inadequately informed about the evidence linking biomarkers to patient outcomes^4^. Immunotherapy is increasingly used for treating cancers, particularly the use of immune checkpoint inhibitors (ICIs) either singly or in combination with traditional chemotherapy drugs, which are recommended for treating approximately 50 tumor types^5^. The successful application of immunotherapy suggests the value of identifying immune-associated biomarkers for further clinical use.

CD74, a type-II transmembrane glycoprotein, is involved in several biological processes. It has been shown to function as a chaperone in the transport of MHC II molecules, which are involved in antigen presentation^6^. Furthermore, CD74 molecules on cell surfaces act as receptors for the macrophage-migration inhibitory factor (MIF)^7^. CD74 expression is elevated in various cancers, including multiple myeloma^8^, invasive pancreatic cancer^9^, colorectal adenoma^10^, high-grade gliomas^11^, non-small cell lung cancer^12^, and advanced melanoma^13^. Increased expression has also been associated with improved outcomes in glioblastoma^14^. However, the function of CD74 in cancer remains poorly understood.

Pan-cancer analysis is valuable for evaluating the functions and underlying molecular mechanisms of specific genes in cancer, allowing potential translation to the clinic^15^. As there is an overall paucity of information on the role of CD74 in cancer and no pan-cancer analysis, we conducted an extensive analysis of the gene using various publicly available databases to determine its expression levels, genomic alterations, and prognostic associations in pan-cancer. CD74 function was also evaluated in terms of DNA damage and repair processes, cancer immunity, and epigenetic modifications. Multiple fluorescence staining showed that CD74 may represent a marker for M1 macrophage infiltration in pan-cancer. Compounds that could activate CD74 in certain cancers were also investigated. The findings enhance our knowledge of the functions of CD74 in various tumors and provide directions for the development of novel treatment strategies.

## Results

### Expression of CD74 in pan-cancer

**Figure 1:**
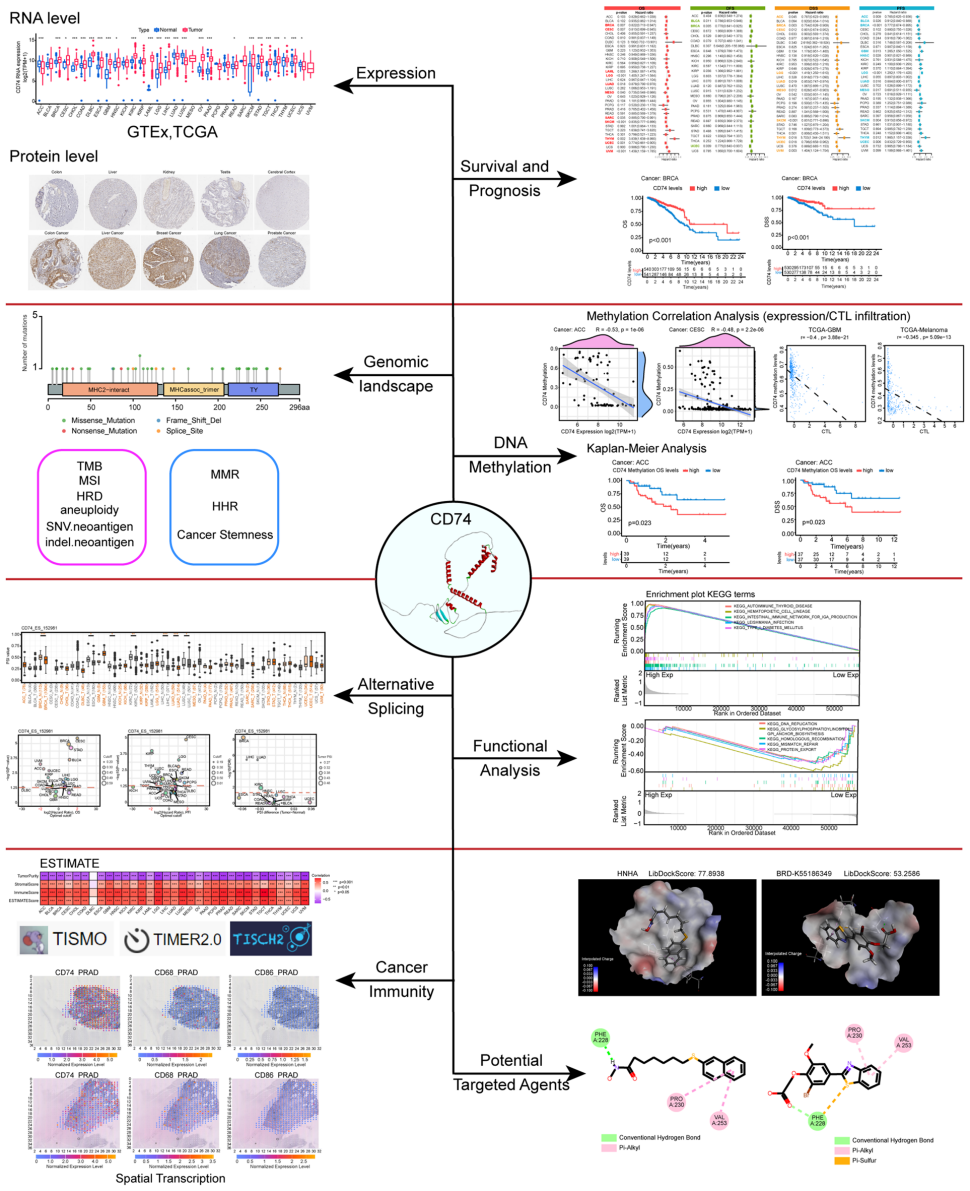
Flow chart of the study.

**Figure 2:**
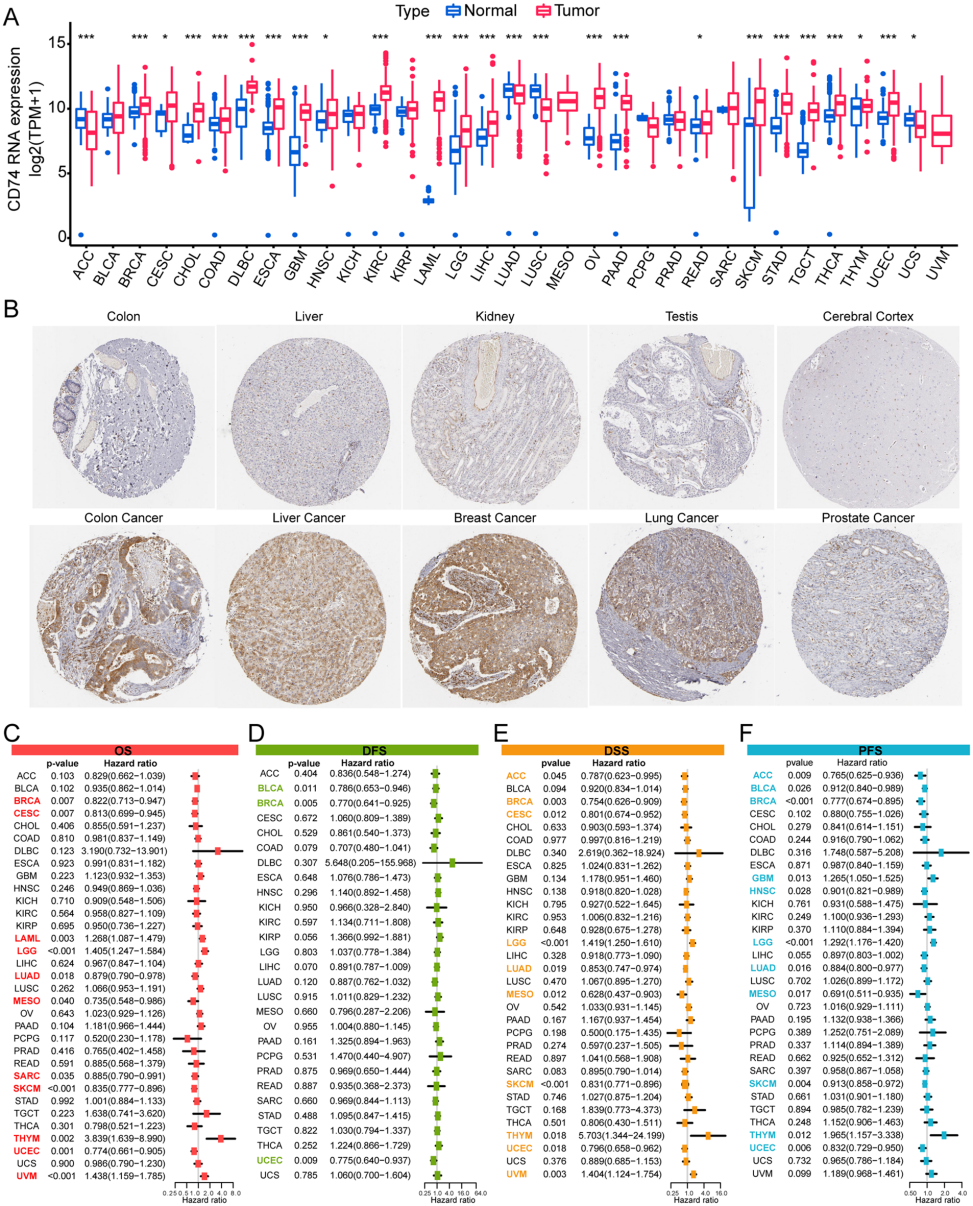
Differential expression of CD74 and its ability to predict survival outcomes in patients. (**A**) CD74 levels in tumor and normal tissue samples from the GTEx and TCGA databases. (**B**) IHC images showing CD74 staining, from the HPA database. (**C**) Forest plot showing associations between CD74 levels and OS. (**D**) Forest plot showing associations between CD74 levels and DFS. (**E**) Forest plot showing associations between CD74 levels and DSS. (**F**) Forest plot showing associations between CD74 levels and PFS. *p < 0.05, **p < 0.01, ***p < 0.001.

Figure 1 illustrates the flow chart of the study. Data on pan-cancer CD74 mRNA levels were obtained from the TCGA and GTEx databases. CD74 mRNA levels were significantly elevated in 21 cancers (BRCA, CESC, CHOL, COAD, DLBC, ESCA, GBM, HNSC, KIRC, LAML, LGG, LIHC, OV, PAAD, READ, SKCM, STAD, TGCT, THCA, THYM, and UCEC) and reduced in ACC, LUAD, LUSC, and UCS (Fig. 2A). CD74 protein levels were then compared between tumor and normal tissues using the HPA database. IHC showed intense CD74 protein staining in COAD, LIHC, BRCA, and LUAD (Fig. 2B). The UALCAN results were similar overall to those of HPA (Fig. S1A). In addition, we obtained the expression distribution of each isoform and isoform usage in CD74 from the GEPIA2 database (Fig. S1B,C). CD74 levels and clinical features were then investigated, finding negative associations between CD74 and high-grade UCEC and BLCA (Fig. S2) and higher CD74 expression in patients with HPV + HNSC, IDHwt-LGG, EBV-STAD, and seminoma-TGCT in comparison with other subtypes (Fig. S2).

### Diagnostic and prognostic value of CD74 across cancers

CD74 was found to be effective (the area under the curve [AUC] > 0.7) in predicting nine cancers, indicating a high diagnostic value (Fig. S3). Investigation of its prognostic value in pan-cancer, four prognostic markers were analyzed in 33 cancer types in relation to OS, DFS, DSS, and PFS. Univariate analysis showed that CD74 levels could significantly predict OS in LAML, LGG, THYM, and UVM, and offered protection in BRCA, CESC, LUAD, MESO, SARC, SKCM, and UCEC (Fig. 2C). Regression analysis of DSS showed that CD74 was a protective in BLCA, BRCA, and UCEC (Fig. 2D), a risk factor for DFS in LGG and THYM, and a protective factor in ACC, BRCA, CESC, LUAD, MESO, SKCM, and UCEC (Fig. 2E). Analysis of PFS showed that CD74 levels were predictive of unfavorable prognosis in GBM, LGG, and THYM but were protective in ACC, BLCA, BRCA, HNSC, LUAD, MESO, SKCM, and UCEC (Figs. 2F, S4–S6). These findings indicate that while CD74 levels were significantly linked to prognosis, the relationships are complex and multifaceted.

### CD74 genetic alterations are associated with genomic instability in pan-cancer

**Figure 3:**
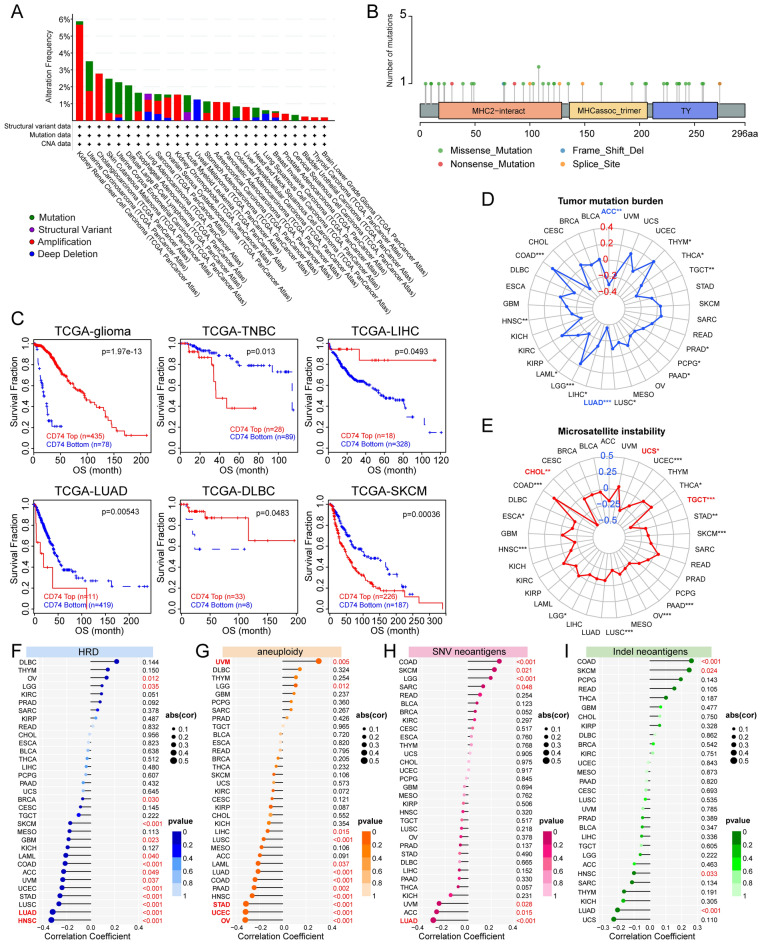
Associations between CD74 and genomic instability. (**A**) Analysis of genomic alterations in CD74, including mutations, amplifications, deep deletions, and structural variants, from TCGA. (**B**) Landscape of CD74 SNVs, including missense and nonsense mutations, frameshift deletions, and splice site variations. (**C**) KM curves showing the prognostic significance of CNVs in CD74. (**D**) Radar charts showing associations between CD74 and TMB. The Blue font indicates |a correlation coefficient| of ≥ 0.3. (**E**) Radar charts showing associations between CD74 and MSI. The red font indicates |a correlation coefficient| of ≥ 0.3. (**F**–**I**) Lollipop graph showing relationships between CD74 and HRD, aneuploidy, SNV neoantigens, Indel neoantigens. Dot size indicates sample size and color denotes p-value. Cancers with |a correlation coefficient|≥ 0.3 are shown in red bold type, with regular red font indicating that the cancer meets the p-value < 0.05 threshold. *p < 0.05, **p < 0.01, ***p < 0.001.

It is well-known that genetic alterations are associated with tumorigenesis. Analysis of CD74 CNV and SNV showed markedly increased amplification of CD74 in KIRC and increased SNV rates in diffuse large B-cell lymphoma and UCS, although an increase in deep deletions was seen (Fig. 3A). Figure 3B illustrates the sites, types, and numbers of CD74 alterations. Analysis of CNVs on TIDE showed longer survival in patients with higher CD74 CNVs in glioma, LIHC, and DLBC but reduced survival in TNBC, LUAD, and SKCM (Fig. 3C). Furthermore, the associations of CD74 with TMB, MSI, HRD, aneuploidy, and neoantigens were investigated, as these are often seen in tumors where they influence both prognosis and response to treatment (Fig. 3D–I)^16^. Interestingly, it was shown that the levels of CD74 expression in LUAD were inversely connected with TMB, HRD, and SNV neoantigens, respectively. This finding raises the possibility that CD74 is involved in HRD and genetic instability related pathways in lung cancer.

### CD74 is associated with DNA repair and stemness in pan-cancer

Both MMR and HRR are responsible for the maintenance of genomic integrity^17^. In addition, regulation of the activities of stem cells is important in both cancer progression and treatment resistance and response^18^. Thus, the associations between CD74 and MMR-related genes, the HRR signature, and stemness were analyzed.

**Figure 4:**
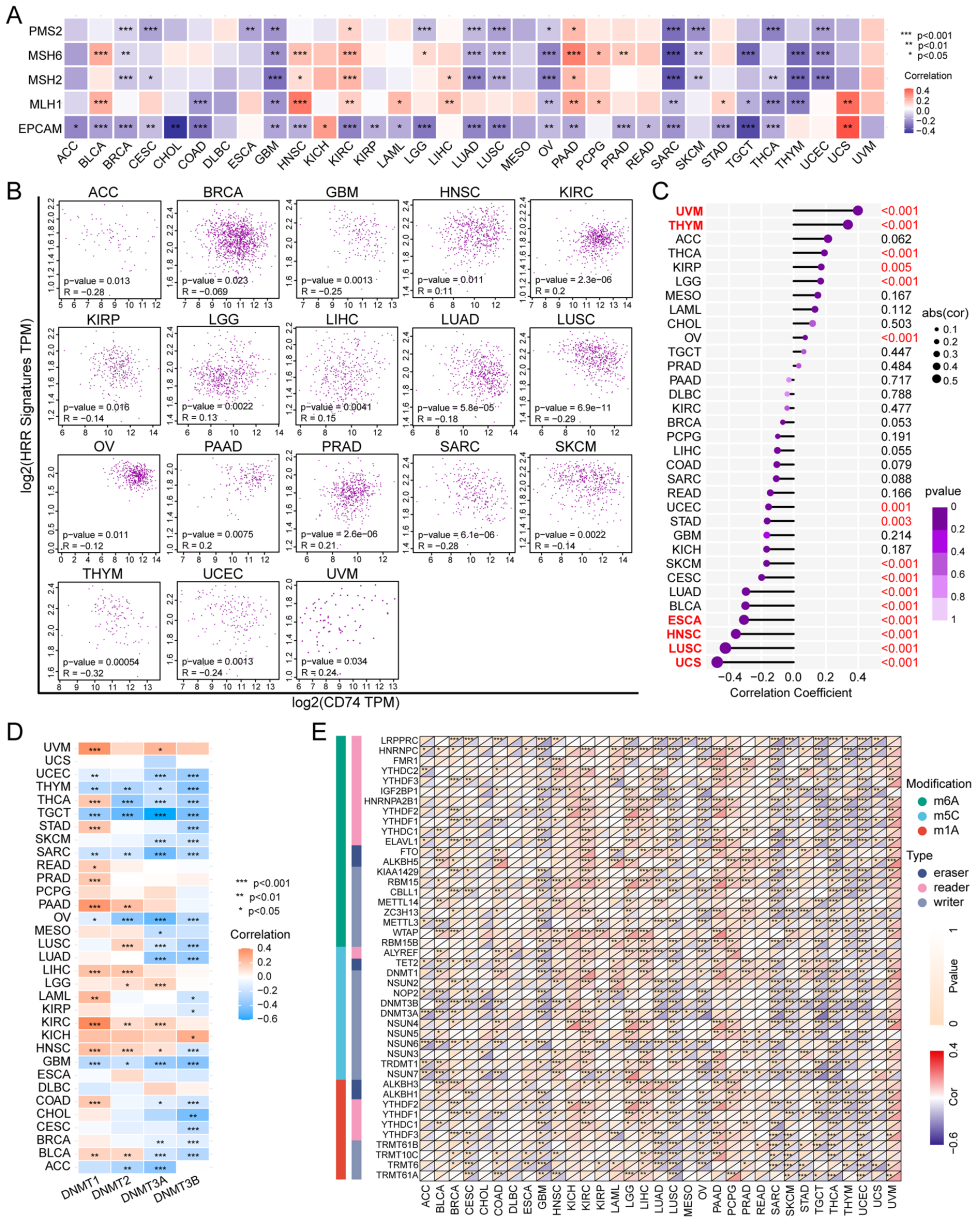
Associations of CD74 with DNA repair, stemness, and epigenetic modifications. (**A**) Heatmap showing relationships between CD74 and five MMR-related genes. (**B**) Correlations between CD74 levels and HRR signature of 30 genes. (**C**) Lollipop graph showing the relationship between CD74 levels and stemness; dot size indicates sample size and color denotes p-value. (**D**) Heatmap showing associations between CD74 levels and four methyltransferases. (**E**) Heatmap showing relationships between CD74 levels and RNA modifications. *p < 0.05, **p < 0.01, ***p < 0.001.

Negative correlations were observed between CD74 and MMR-related genes in many cancer types, including BRCA, CESC, GBM, LUAD, LUSC, OV, SARC, TGCT, THCA, THYM, and UCEC (Fig. 4A), as well as with the HRR signature in ACC, GBM, LUSC, SARC, and THYM (Fig. 4B) and with stemness in UCS, LUSC, HNSC, and ESCA (Fig. 4C).

### Analysis of epigenetic alterations of CD74 in pan-cancer

Tumors are known to use epigenetic mechanisms, such as DNA methylation, for immune evasion^19^. Thus, methylation in the CD74 promoter region was examined, observing that CD74 methylation was negatively associated with its mRNA expression level (Fig. S7A). Relationships between CD74 and the levels of the four methyltransferases (DNMT1, DNMT2, DNMT3A, DNMT3B) were then evaluated (Fig. 4D). This showed close correlations between CD74 levels and the expression of at least one methyltransferase in the majority of cancer types, apart from DLBC, ESCA, PCPG, and UCS. Associations between CD74 methylation and CTLs were also investigated using TIDE, with negative correlations seen in GBM, melanoma, TNBC, LIHC, and LUAD (Fig. S7B). Furthermore, relationships between CD74 methylation and survival prediction (OS, DSS, DFS, and PFS) were examined by KM curves for all 33 cancer types (Fig. S8), showing that reduced methylation was predictive of longer survival in ACC and BLCA.

Dysregulation of pathways associated with RNA modification, including m1A, m5C, and m6A modifications, has also been linked to tumorigenesis and cancer progression^20^. Associations between CD74 levels and those of 44 regulators of RNA modifications, specifically, methyltransferases (writers), demethylases (erasers), and RNA-binding proteins (readers). Surprisingly, CD74 levels were found to be positively associated with m1A, m5C, and m6A methylation in KICH, PCPG, and UVM but negatively linked with all methylation types in ACC, CESC, CHOL, GBM, LUSC, OV, SARC, TGCT, THCA, THYM, and UCEC (Fig. 4E). These results suggest the involvement of both DNA methylation and mRNA modification of CD74 in various cancers.

### Alternative splicing of CD74 and survival outcomes

**Figure 5:**
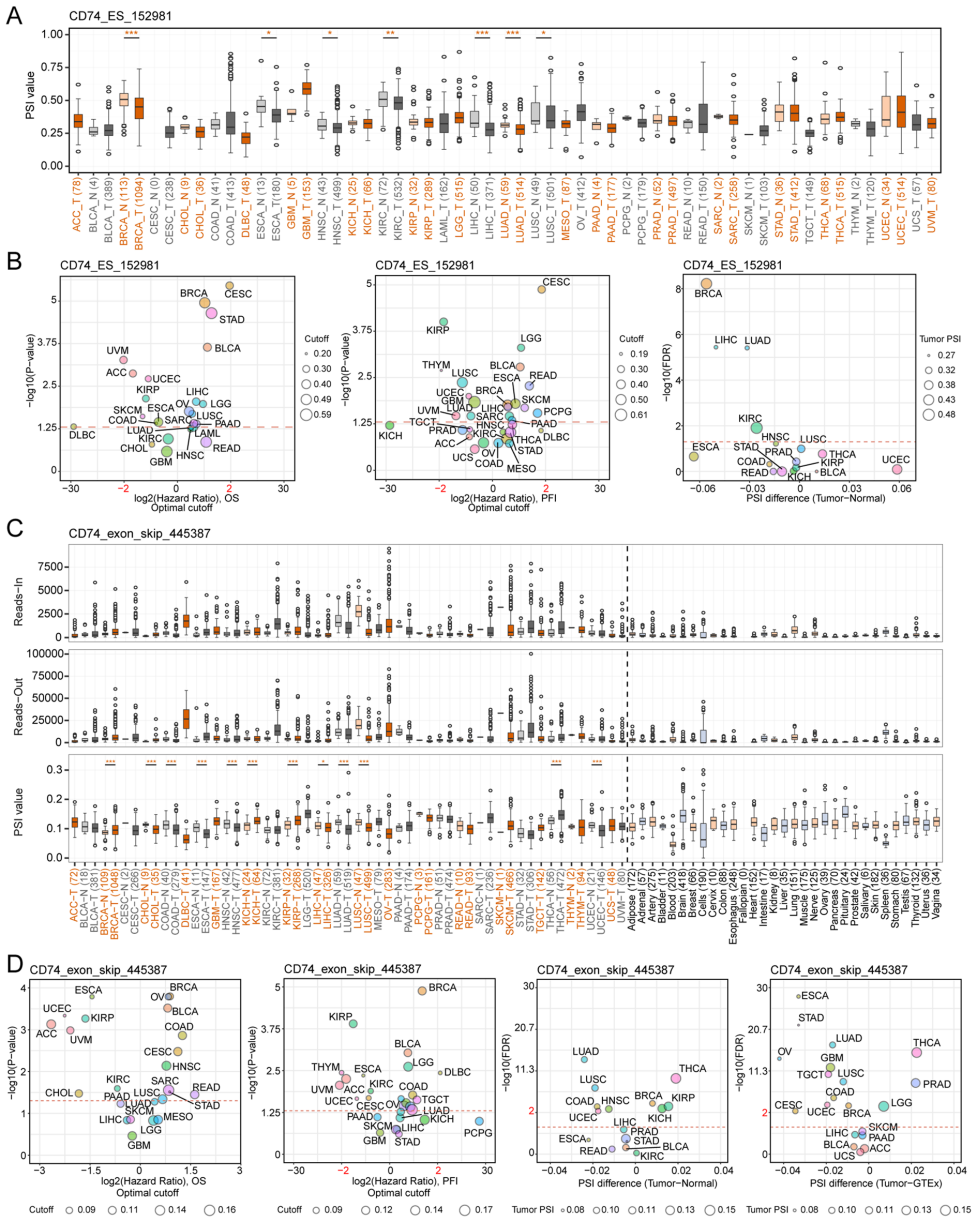
Associations between alternative splicing of CD74 and patient prognosis. (**A**) PSI values of CD74_ES_152981 in tumor and normal samples. Colors indicate different tumors (T) and adjacent normal tissues (N). (**B**) The reads-in, reads-out, and PSI values of CD74_exon_skip_445387 in tumor and normal tissues, respectively. Colors indicate tumor (T) and corresponding adjacent (N) tissues, with black indicating normal tissues. (**C**) PSI values in tumor and normal tissues and association between differences between CD74_ES_152981 events and prognosis (OS, PFS). (**D**) PSI values in tumor and normal tissues and association between CD74_exon_skip_445387 events and prognosis. *p < 0.05, **p < 0.01, ***p < 0.001.

AS results in the production of different transcripts and proteins or noncoding RNAs from a gene. CD74 AS was examined using OncoSplicing, resulting in the identification of 37 clinically relevant AS events (Table S2), although we have concentrated on the CD74_ES_152981 event in the TCGA SpliceSeq database and the CD74_exon_skip_445387 event in the TCGA SpIAdderSeq database here. Figure 5A illustrates the PSI values of the CD74_ES_152981 event in pan-cancer. Lower PSI was seen in BRCA, ESCA, HNSC, KIRC, LIHC, LUAD, and LUSC relative to normal samples. PSI values of the CD74_exon_skip_445387 event are shown in Fig. 5B. While reduced PSI was seen in eight tumor types, opposite results were found in BRCA, KICH, KIRP, and THCA. Additionally, Fig. 5C and D summarize the differences in PSI between tumor and normal samples, as well as relationships between CD74_ES_152981, CD74_exon_skip_445387, and prognosis (OS, PFS). The findings suggest the significance of regulated CD74 AS events in cancer progression.

### CD74 is involved in cancer immune pathways in BRCA

**Figure 6:**
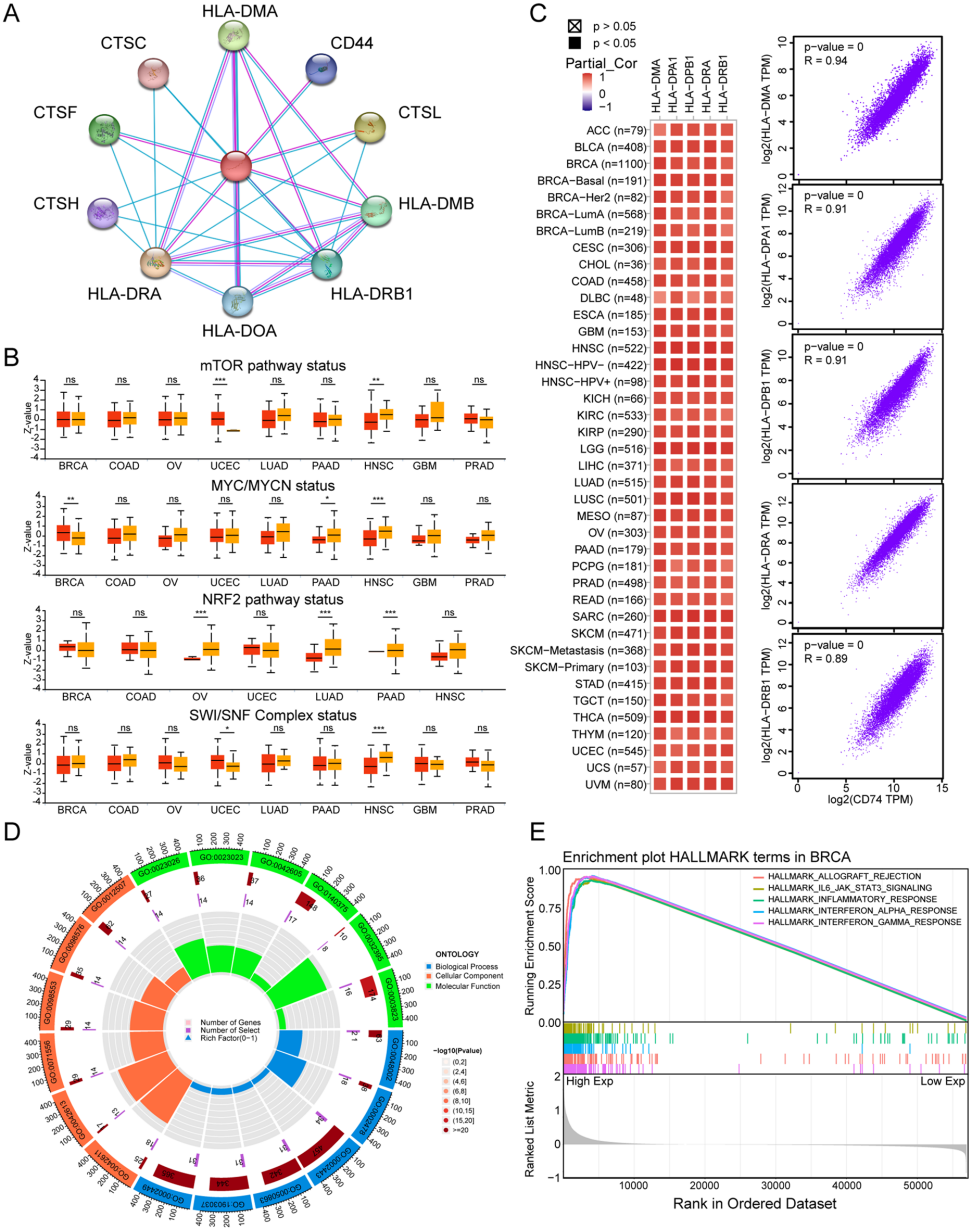
Involvement of CD74 in cancer immunity. (**A**) PPI network of CD74 and experimentally verified binding partners. (**B**) Boxplots showing CD74 levels in somatically altered and unaltered pathways, from UALCAN. (**C**) Associations between CD74 and the top five co-expressed genes for individual tumors (left) and all tumor samples (right). (**D**) Enriched GO pathways of the top 100 genes co-expressed with CD74. (**E**) Enriched HALLMARK terms in BRCA, assessed by GSEA. The groups were defined according to the median CD74 level. ns, p ≥ 0.05, *p < 0.05, **p < 0.01, ***p < 0.001.

CD74 functions in tumorigenesis and their underlying mechanisms were further investigated. Figure 6A shows the top 10 experimentally verified interacting proteins in the network compiled by STRING. We then noticed that CD74 expression was increased in UCEC patients with somatic alterations of the mTOR pathway or the SWI/SNF complex status, while CD74 expression was decreased in HNSC patients with somatic alterations of the mTOR pathway, the MYC/MYCN pathway or the SWI/SNF complex status (Fig. 6B). The correlation between CD74 expression and these pathway-related signatures was also explored (Fig. S9). The results suggested that CD74 is potentially involved in multiple oncogenic pathways. The top 100 genes found to be co-expressed with CD74 were examined using GEPIA2.0, finding that the top five genes (HLA-DMA, HLA-DPA1, HLA-DPB1, HLA-DRA, and HLA-DRB1) were highly correlated with CD74 in most tumor types (Fig. 6C). GO analysis of the top 100 genes indicated the involvement of CD74 in immune-related pathways such as Following this, GO enrichment analyses were conducted using the top 100 co-expressed genes antigen processing and presentation (Fig. 6D, Table S3). The Hallmark results for BRCA suggested a close relationship between the IL6-JAK-STAT pathway and CD74 (Fig. 6E, Table S4).

### CD74 and immune infiltration

**Figure 7:**
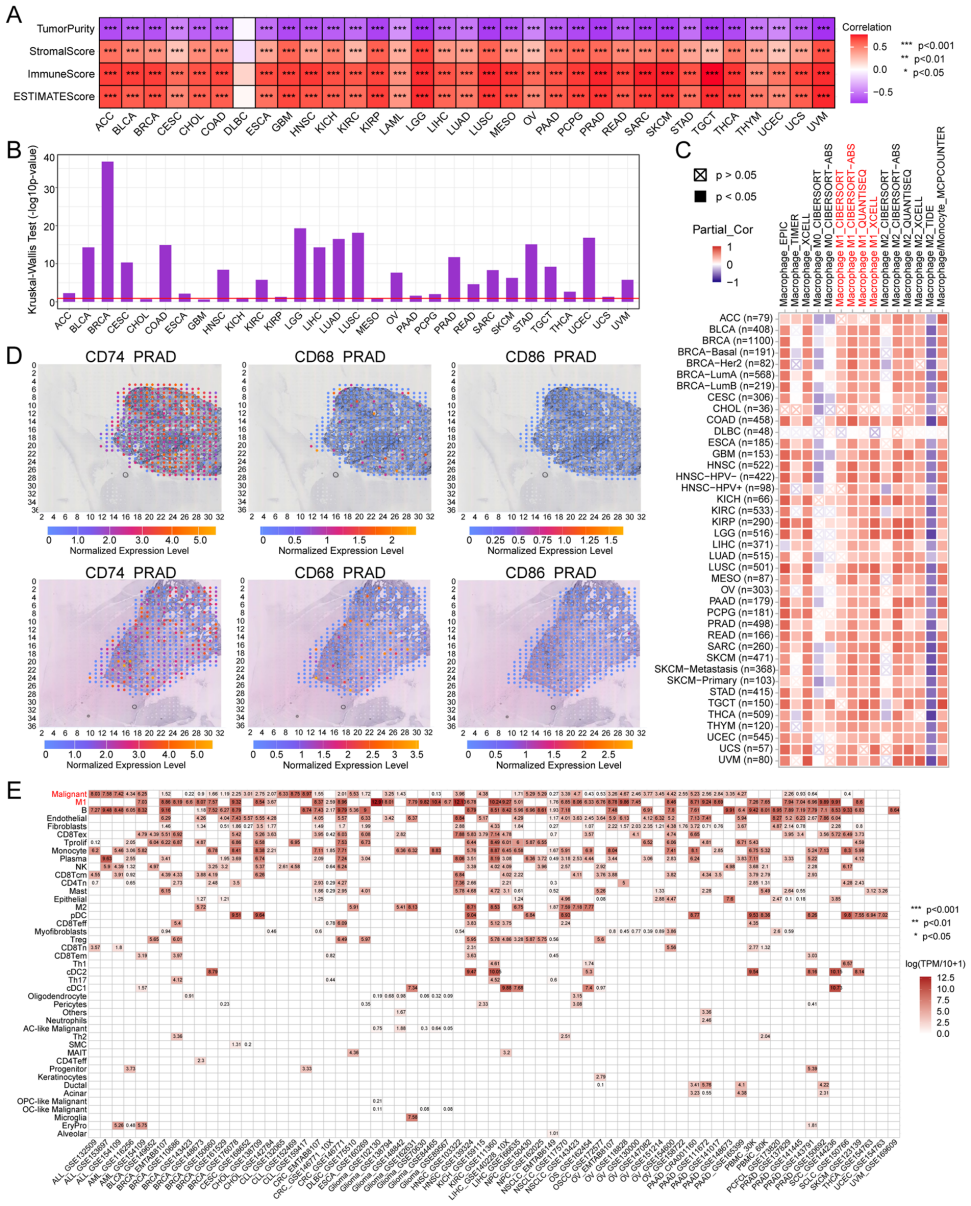
CD74 is a potential marker of M1 macrophage infiltration. (**A**) Heatmap showing relationships between CD74 and ESTIMATEScore, ImmuneScore, StromalScore, and tumor purity. (**B**) Associations between CD74 and immune subtypes, from TISDB. (**C**) Infiltration by M1 macrophages, calculated by multiple algorithms in TIMER2.0. Partiall_Cor indicates partial correlation. (**D**) Spatial distribution of CD74, CD68, and CD86 expression. Dot colors indicate level of expression. (**E**) CD74 levels in single-cell tumor clusters, from the TISCH online tool. *p < 0.05, **p < 0.01, ***p < 0.001.

The ESTIMATE algorithm was used to explore the associations between CD74 and the tumor microenvironment (TME) by calculating the ImmuneScore, StromalScore, and ESTIMATEScore in 33 tumor types. It was found that there was a positive correlation between CD74 and the ImmuneScore in most cancers (Fig. 7A), with strong correlations seen in the top six tumor types, as seen in Fig. S10A. Differential expression of CD74 was then investigated in different immune subtypes using TISDB. The histogram in Fig. 7B illustrates the significant association between CD74 levels and immune subtypes in 27 cancers (27/33, 81.8%), with the top six shown in Fig. S8B. Relationships between CD74 and immune-associated genes encoding immunosuppressive and activating proteins, chemokines and their receptors, and MHC proteins, were then explored (Fig. S11). Investigation of the differences in CD74 levels before and after in vitro cytokine treatment (Fig. S12A) and before and after in vivo anti-PDL1 or anti-CTLA4 treatment (Fig. S12B) using the web tool TISMO showed increased CD74 levels after all these treatments. All these findings indicate that CD74 may be involved in the regulation of immune cell infiltration and the functions of TME-associated genes in most cancers. Further analysis of the associations between CD74 and 14 functional cancer states using pan-cancer data from CancerSEA (Table S5), with a specific focus on BRCA, indicated a positive association with both angiogenesis and inflammation (Fig. S13A), suggesting the involvement of CD74 in these pathways in BRCA.

### CD74 is a potential marker of M1 macrophage infiltration in pan-cancer

**Figure 8:**
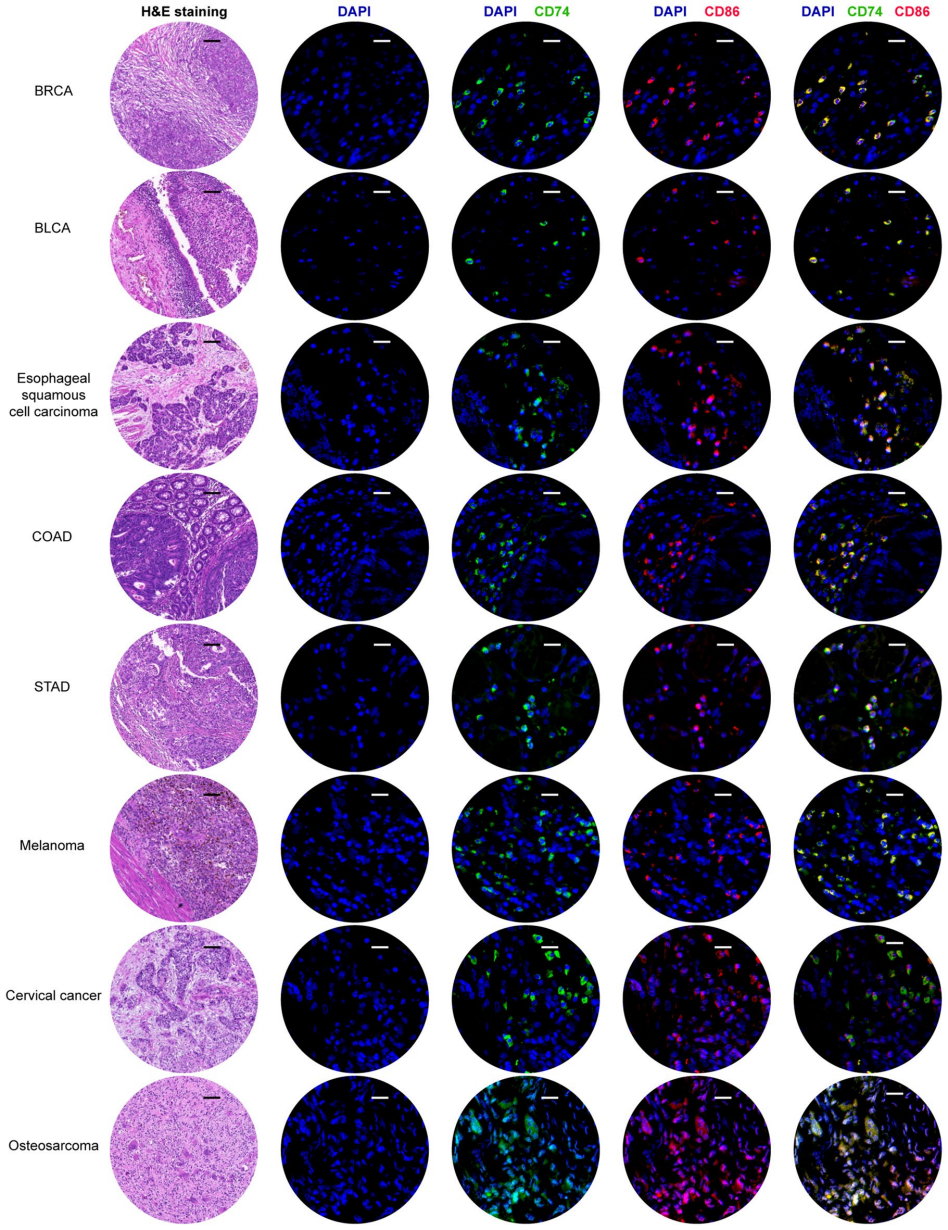
H&E and multiple fluorescence staining of CD74 in pan-cancer tissue chips. Nuclei are stained blue and CD86 and CD74 are stained red and green, respectively. Scale bar in H&E staining represents 100 μm. Scale bars in multiple fluorescence staining represent 20 μm.

TIMER2.0 was used to determine associations between CD74 and immune cell levels. This showed a significant positive correlation with the contents of M1 macrophages in 32 cancers, with the exception of DLBC (Fig. 7C), suggesting that CD74 may represent a marker for infiltration by M1 macrophages in pan-cancer. SpatialDB was used to determine the overlap between the distributions of CD74, the macrophage marker CD68, and the M1 macrophage marker CD86 in PRAD and melanoma tissues (Figs. 7D, S13B). This showed that the distributions of CD74 and CD86 were similar, suggesting possible co-expression of the genes. Single-cell CD74 expression data were obtained from TISCH (Fig. 7E), demonstrating that CD74 was mostly found in both M1 macrophages and tumor cells in most cancers. For verification, tissue sections were examined by fluorescence staining, showing co-expression of CD74 and CD86 in eight cancer types (BRCA, BLCA, esophageal squamous cell carcinoma, COAD, STAD, melanoma, cervical cancer, and osteosarcoma) (Fig. 8). These data indicate the close associations between CD74 levels and the infiltration of M1 macrophages, suggesting that CD74 may be a tumor-specific biomarker.

### Effects of CD74 on treatment response and investigation of compounds activating CD74

**Figure 9:**
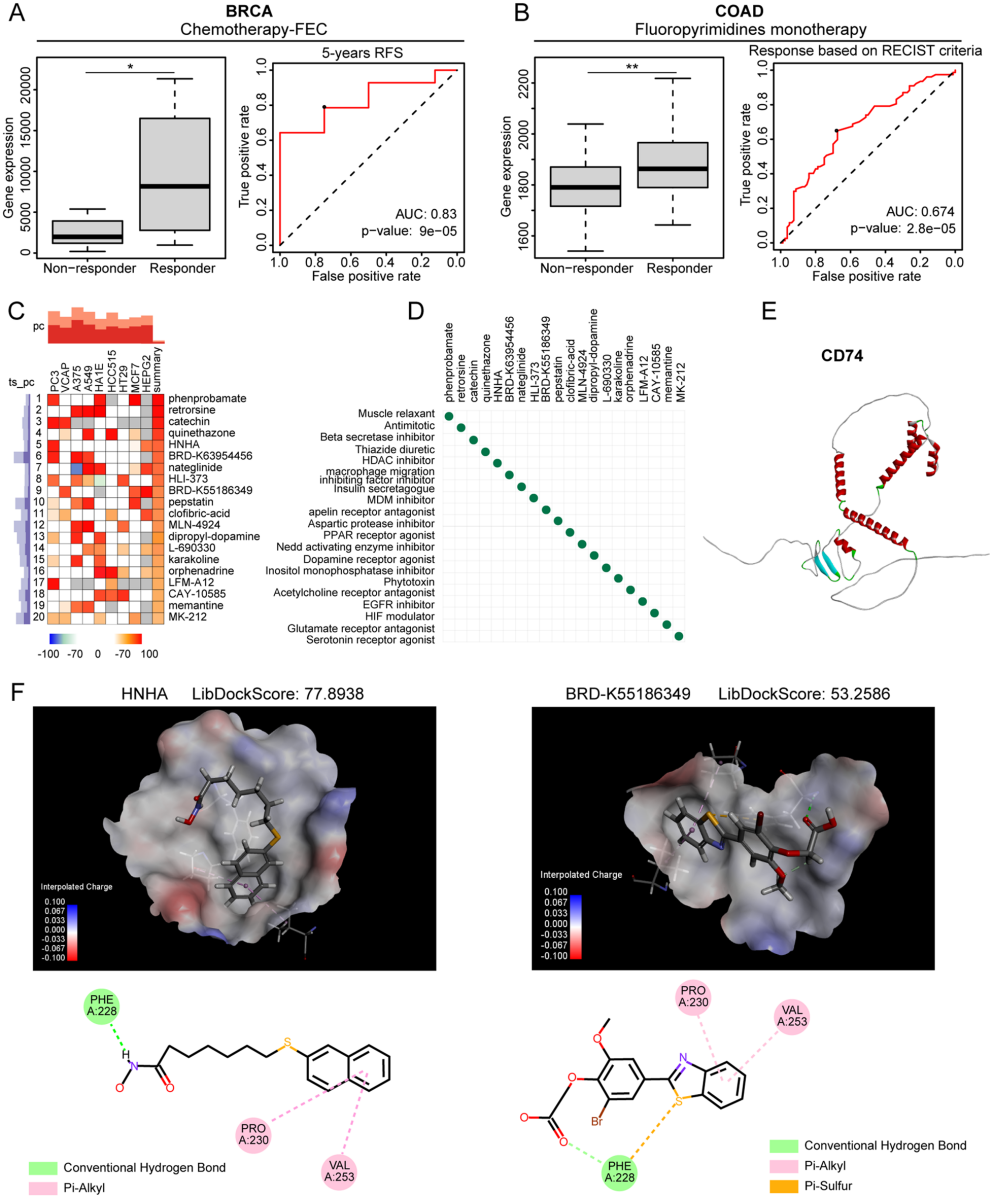
Prediction of therapeutic response by CD74 and docking with activating compounds. (**A**) Boxplots comparing CD74 levels between BRCA patients who responded or did not respond to treatment; ROC curves showing the accuracy of CD74 levels in predicting the patient response, from the ROCplotter website. (**B**) Boxplots comparing CD74 levels between COAD patients who responded or did not respond to treatment; ROC curves showing the accuracy of CD74 levels in predicting patient response. (**C**) Heatmap showing top 20 compounds leading to transcriptional changes. Colors indicate similarity scores. (**D**) Scatterplots showing MoA values of top 20 compounds. (**E**) Model of CD74 by AlphaFold2.0. (**F**) 3D model of CD74 structure showing drug-binding pocket. 2D graphs below show 2D structures of drugs, their interactive amino acids, binding forces, and spatial distances. *p < 0.05, **p < 0.01, ***p < 0.001.

Data from ROC Plotter were analyzed to determine associations between CD74 and therapeutic outcomes. High levels of CD74 were seen in BRCA patients who responded to chemotherapy, especially treatment with fluorouracil, epirubicin, and cyclophosphamide combinations, with an AUC of 0.83 for 5-year recurrence-free survival (Fig. 9A). Similar results were seen in COAD patients following fluoropyrimidine monotherapy, with an AUC of 0.67 based on RECIST criteria (Fig. 9B).

Given the poor outcome of conventional treatment in BRCA patients with low CD74 expression, we sought to identify drugs that could potentially activate CD74 and improve the sensitivity of cancer to current chemotherapy. Filtering compounds using the cMap tool caused the same transcriptional alterations as increased CD74 expression in nine cancer cell lines and identified the top 20 compounds predicted to activate CD74 (Fig. 9C). The mechanism of action (MoA) of these 20 compounds is shown in Fig. 9D. To further screen for small-molecule drugs that can bind to CD74, we performed molecular docking between the top 20 candidate small-molecule drugs and CD74. Five models of CD74 were constructed using alphaFold2.0 from the sequences shown in Supplementary Material 1, and the rank_1 model was selected (Fig. 9E) for molecular docking using Discovery Studio v19.1.0. The compounds HNHA and BRD-K55186349 docked successfully with CD74 with LibDockScores of 77.8938 and 53.2586, respectively. Figure 9F shows the 3D structures of the interaction sites, together with the interactive forces and distances in the 2D graph. Thus, HNHA and BRD-K55186349 were identified as possible activators of CD74 in BRCA, suggesting their potential use together with chemotherapy drugs to enhance treatment sensitivity, especially that of combined fluorouracil, epirubicin, and cyclophosphamide treatment.

## Discussion

The type II transmembrane protein CD74 has been found to be abnormally upregulated in a variety of cancers. It has also been proposed to be predictive of both metastasis and clinical outcomes in various cancers^14,21^. Inhibition of MIF1/MIF2 reduces the tumor burden of bladder cancer in preclinical models, an effect that was found to be accomplished in part through activation of CD74, suggesting the significance of CD74 in tumor development^22^. However, there is minimal information on its function in other cancers. Thus, we conducted a comprehensive analysis of the characteristics, clinical relevance, and potential functions of CD74 in tumor immunity, as well as screening for potential activating drugs in pan-cancer.

Overexpression of CD74 was observed in 21 cancers compared with normal tissues. Negative associations were seen between CD74 and high-grade BRCA and UCEC, suggesting the possibility that increased CD74 levels may hinder cancer progression. Regression and KM analyses of prognosis prediction in pan-cancer showed that increased expression of CD74 was associated with prolonged survival in patients with BRCA, CESC, LUAD, MESO, SARC, SKCM, and UCEC, suggestive of a protective role in these cancers. These findings are consistent with those of other studies^21^. Thus, CD74 may be important in the survival and outcome prediction in patients with cancer, suggesting its promise as a potential biomarker.

Many patients have ‘cold” tumors that respond poorly to present treatments with checkpoint inhibitors^23^. Reversal of this “cold” state requires the use of multiple forms of treatment. Previous studies have shown that the expression of CD74 on immune cells modulates the activity of cancer cells. Wang et al. performed scRNA-seq on an undifferentiated carcinoma with osteoclast-like giant cells (OGCs) of pancreas (UCOGCP) patient^24^. CellphoneDB showed strong CD74-MIF, CD74-COPD and CD74-APP receptor-ligand interactions between OGCs and macrophages_I/III^24^. In addition, Figueiredo et al. found that blocking MIF-CD74 signaling on macrophages and dendritic cells restored anti-tumor immune responses against metastatic melanoma^25^. In our study, CD74 expression was found to be increased following cytokine treatment in several cancers, as well as following ICI treatment (anti-PD1, anti-PDL1, and anti-CTLA4), strongly suggesting that CD74 activates the immune response in most cancers.

Our study paves the way for future experimental studies. There is an imbalance between M1 and M2 macrophages in many cancers. This skewed ratio is thought to contribute to tumor development. M1 macrophages have "killing" activity and are frequently linked to an anti-tumor response^26^. Thus, activation of M1 macrophages is a potential therapeutic strategy. In our study, CD74 was shown to be a marker for the infiltration of M1 macrophages in pan-cancer by bulk, spatial, single-cell transcriptional analyses along with immunofluorescence, suggesting that CD74 has potent clinical translational capabilities and may be used in combination with ICIs to reverse “cold” tumors. Moreover, GSEA findings indicate that elevated CD74 expression is linked to the activation of a range of immune responses in breast cancer, including IFN-α and IFN-γ responses, and the IL6-JAK-STAT3 pathway. Future experimental studies could focus on these three aspects to investigate the downstream molecular mechanisms of CD74 in breast cancer. Notably, we investigated and identified two potential CD74 activators (HNHA and BRD-K55186349). Future studies could investigate whether these agents activate CD74 to enhance sensitivity to conventional chemotherapy in chemoresistant BRCA patients.

## Conclusion

This is the first analysis of CD74 in pan-cancer. It was found that CD74 is expressed differentially between tumor and normal tissues, suggesting its potential as an independent predictor of outcomes in many cancer types. However, further investigation is needed into the specific functions of CD74 in individual tumor types. CD74 levels were also associated with TMB, MSI, HRD, aneuploidy, neoantigen, DNA repair, cancer stemness, DNA methylation, and chemoresistance. Significantly, CD74 was found to be associated with cancer immunity and acted as a biomarker of the infiltration of M1 macrophages. We also identified potential small-molecule compounds interacting with CD74 that could be used as novel treatments. These results enhance our understanding of the function of CD74 in various cancers and provide new directions for patients who respond poorly to current therapies.

## Materials and methods

### Pan-cancer data collection and processing

Clinical and expression data on CD74 in pan-cancer and normal tissues were collected from the TCGA and GTEx databases and were analyzed with UCSC Xena^27^. Gene expression was converted to transcripts per million (TPM) and log-transformed (log_2_(TPM + 1)). Table S1 shows the abbreviations for the different cancer types. Immunohistochemistry (IHC) images of CD74 in tumor and normal tissues were obtained from the Human Protein Atlas (HPA)^28^. Processed pan-cancer simple-nucleotide variation (SNV) data were obtained from cBioPortal^29^ together with merged HM27 and HM450 methylation data. CD74 protein sequences in the FASTA format were downloaded from NCBI Protein.

### Analysis of CD74 in pan-cancer

The expression of CD74 in 33 tumor types together with their paired normal tissues was analyzed using the GTEx-TCGA cohort. Boxplots were constructed using the “limma” and “ggplot2” packages in R^30^, showing associations between CD74 mRNA levels and clinicopathological characteristics (subtypes and TNM stages). Data from the Clinical Proteomic Tumor Analysis Consortium (CPTAC), obtained from The University of Alabama at Birmingham Cancer Data Analysis Portal (UALCAN)^31^, were used for the comparison of CD74 protein levels between tumor and normal tissues. The expression distribution and the isoform usage of each isoform in CD74 were obtained from the GEPIA2 database^32^.

### Prognostic and diagnostic analysis of CD74

Receiver operating characteristic (ROC) curves, constructed by the “pROC” R package, and the areas under the curve (AUCs) were used to assess the diagnostic significance of CD74 in pan-cancer. Four clinical outcomes, namely, overall survival (OS), disease-specific survival (DSS), disease-free survival (DFS), and progression-free survival (PFS) were used in analyzing the prognostic utility of CD74 using univariate Cox regression with the “survival” and “forestplot” packages in R. Variables were assessed in terms of the hazard ratio (HR), 95% confidence interval, and p-value, with p < 0.05 considered statistically significant. Kaplan–Meier (KM) curves were used to assess the relationship between CD74 levels and prognosis (using the median expression as the threshold) using the R packages "survive" and "survminer".

### Genomic alteration and genetic heterogeneity analysis of CD74

Genetic alterations in CD74 were analyzed using cBioPortal. The R package "maftools" was used to display the mutational landscape of CD74^33^. Survival curves for CD74 copy number variants (CNVs) were obtained from the Copy_Number module of the Tumor Immune Dysfunction and Exclusion (TIDE) to assess the prognostic association of CNVs^34^. Tumor mutation burden (TMB) values were assessed using “maftools” in R and information on microsatellite instability (MSI), homologous recombination deficiency (HRD), aneuploidy, and neoantigens (including neoantigens resulting from SNVs and indels) for specific tumor types was derived from earlier studies^35,36^. Associations between CD74 levels and TMB, MSI, HRD, aneuploidy, and neoantigens were evaluated.

### Association of CD74 with DNA repair, and cancer stemness

Pan-cancer associations between the levels of CD74 and five mismatch repair (MMR) genes were examined^37^. For determination of the pan-cancer homologous recombination repair (HRR) signature, 30 genes associated with HRR identified from the ARIEL3 clinical trial^38^ were evaluated using GEPIA2^32^ to assess their relationships with CD74. Tumor stemness scores were assessed using the OCLR algorithm to calculate gene methylation in various tumors^39^ and their relationships to CD74 levels were examined.

### The analysis of CD74 correlation with DNA methylation and mRNA modification

Associations between CD74 levels and four DNA methyltransferases were assessed^40^ using Spearman correlations. Relationships between methylation of the CD74 promoter region and cytotoxic T lymphocytes (CTLs) were determined using the Methylation module in TIDE^34^ and associations between CD74 methylation and prognosis were examined by KM curves using the “survival” package in R. A heatmap was used to visualize the associated between CD74 and 44 genes responsible for N1-methyladenosine (m1A), 5-methylcytosine (m5C), and N6-methyladenosine (m6A) modifications in pan-cancer^41^.

### Clinically relevant alternative splicing of CD74

Alternative splicing (AS) of CD74 in relation to clinical prognosis was examined using the ClinicalAS module in OncoSplicing, with data from the SplAdder and SpliceSeq projects^42^. PanPlot was used to show the percent spliced-in (PSI) values of tumors and tissues. PSI differences associated with AS (if found in > 3 tumors) were shown in PanDiff plots.

### Genes co-expressed with CD74 and their functional enrichment

A CD74 Protein–Protein Interaction (PPI) network based on experimental verification was constructed using the STRING database^43^. CD74 levels were compared between patients in whom oncogenic pathways had been altered and controls using UALCAN, while correlations between CD74 levels and oncogenic pathway-associated signatures were determined by GEPIA2^44^. The top 100 genes co-expressed with CD74 in pan = cancer were identified using the Similar Gene Detection function in GEPIA2^32^. A heatmap was used to visualize the association between CD74 and the top five co-expressed genes using TIMER 2.0^45^ and scatter plots in GEPIA2. The top 100 genes, identified as having a false discovery rate (FDR) < 0.05 were subjected to GO enrichment analysis using the R package “clusterProfiler”, with retrieval of annotations by “org.Hs.eg.db” in R^46^. Tumor samples were allocated to high- and low-CD74 groups based on the median level of CD74 and the gene set h.all.v7.4.symbols.gmt was obtained from the Molecular Signatures Database for GSEA Hallmark pathway analysis^47^.

### CD74 in the immune microenvironment

ESTIMATE scores, namely, the ImmuneScore, StromalScore, and ESTIMATEScore, were determined for each sample using the “Estimate” package in R^48^. CD74 levels in relation to six immune subtypes (C1: Wound healing; C2: IFN-γ dominant; C3: Inflammatory; C4: Lymphocyte depleted; C5: Immunologically quiet; C6: TGF-β dominant) were examined using the TISDB Subtype module^49^. Heatmaps were compiled to visualize associations between CD74 and immune genes, such as immunostimulatory and immunosuppressive genes, MHCs, chemokines, and chemokine receptors. TIMER 2.0 was used to examine associations between immune cell infiltration and CD74. To examine the effects of cytokine treatment, gene expression was analyzed before and after cytokine, anti-PDL1, and anti-CTLA4 treatment using the Tumor Immune Syngeneic MOuse (TISMO) web tool^50^. Spatial distribution and overlap of CD74 with the macrophage marker CD68 and the M1 macrophage marker CD86 were examined by SpatialDB^51^. The Tumor Immune Single-cell Hub (TISCH) analyzed CD74 levels in different cell types in pan-cancer^52^. Correlations between CD74 and 14 functional cancer statuses were determined from single-cell sequencing data using the “correlation plot” module of CancerSEA^53^.

### Hematoxylin and eosin (H&E) staining of pan-cancer tissue chip

Formalin-fixed, paraffin-embedded pan-cancer tissue sections were from the Department of Pathology, Shanxi Cancer Hospital. All patients provided informed consent and the institutional review board of the hospital approved the use of tissues obtained from tumor resection. We confirmed that all experiments were performed in accordance with relevant guidelines and regulations. Patients had no history of autoimmune disease and had not been treated with radiotherapy or chemotherapy prior to surgery. Tissue sections were stained with hematoxylin and eosin (G1005, Servicebio, China).

### Multiple fluorescence staining

Tissue sections were multiple-fluorescence-stained to verify the ability of CD74 to function as a marker of M1 macrophages. Ten tumor types were investigated. After dewaxing and blocking (5% BSA), the sections were incubated with two primary antibodies, namely mouse anti-CD74 (1:250, ab108393) and rabbit anti-CD86 (1:100, MA1-10293) followed by secondary antibodies (BA1031, BA1105, Boster, Wuhan, China). After counterstaining the nuclei with DAPI, the sections were mounted in an anti-fade mountant and examined and imaged under a confocal microscope (Panoramic MIDI, 3DHistech, Hungary). The excitation and emission wavelengths used were used to obtain multispectral images of the stained sections. For fluorescence spectra, the excitation wavelengths used were DAPI (blue, 330–380 and 420 nm), CY3 (red, 510–560 and 590 nm), and FITC (green, 465–495 and 515–555 nm). Positively stained cells were analyzed using Caseviewer (C.V. 2.4).

### Identification of potential small-molecule drugs and molecular docking

Differences in CD74 between patients who responded to therapy and those who did not were assessed using ROC plotter^54^. Compounds activating CD74 were identified using the “query” tool in cMap^55^. Patients were allocated to high and low CD74 expression groups according to the median of CD74 expression, and genes expressed differentially (DEGs) between the two groups were identified. The top 100 genes in both the upregulated and downregulated categories were used for drug screening. The 20 most significant candidate compounds were shown in heatmaps, together with their mechanisms of action (MoA). To investigate interactions between the compounds and CD74, the CD74 structure was modeled using AlphaFold2 and docked using Discovery Studio v19.1.0^56^ with LibDock after preparation of the molecules and identification of all conformations. Sites and conformations with the highest LibDockScore were used to represent the final binding. Three-dimensional views of the binding pocket and two-dimensional intermolecular force distances were visualized.

### Statistical analysis

Inter-group differences were assessed by two-tailed t-tests or one-way analysis of variance (ANOVA). Data are presented as means ± standard deviations (SDs). Survival significances were assessed using log-rank tests. Pearson’s correlations were used for all correlations, with |r|= 0.3 being considered indicative of a relevant correlative relationship. Differences were considered statistically significant when P < 0.05 and are reported as follows: *P < 0.05, **P < 0.01, ***P < 0.001.

### Ethics approval and consent to participate

This study was approved by the institutional review board of Shanxi Cancer Hospital (Shanxi, China) [2021JCII07]. We confirmed that all experiments were performed in accordance with relevant guidelines and regulations.

### Supplementary Information

Supplementary Figures.

Supplementary Information.

Supplementary Tables.

## Data Availability

The datasets used in this paper are available online, as described in the Methods section.

## Abbreviations

ICIs: Immune checkpoint inhibitors
MIF: Macrophage-migration inhibitory factor
TPM: Transcripts per million
IHC: Immunohistochemistry
HPA: The Human Protein Atlas
SNV: Simple-nucleotide variation
CPTAC: The Clinical Proteomic Tumor Analysis
UALCAN: The University of Alabama at Birmingham Cancer Data Analysis Portal
ROC: Receiver operating characteristic
AUC: Areas under the curve
OS: Overall survival
DSS: Disease-specific survival
DFS: Disease-free survival
PFS: Progression-free survival
HR: Hazard ratio
KM: Kaplan–Meier
CNVs: Copy number variants
TIDE: The tumor immune dysfunction and exclusion
TMB: Tumor mutation burden
MSI: Microsatellite instability
HRD: Homologous recombination deficiency
MMR: Mismatch repair
HRR: Homologous recombination repair
CTLs: Cytotoxic T lymphocytes
m1A: N1-methyladenosine
m5C: 5-Methylcytosine
m6A: N6-methyladenosine
AS: Alternative splicing
PSI: Percent spliced-in
PPI: Protein–protein interaction
FDR: False discovery rate
TISMO: The tumor immune syngeneic mouse
TISCH: The tumor immune single-cell hub
H&E: Hematoxylin and eosin
MoA: Mechanisms of action
ANOVA: One-way analysis of variance
SDs: Standard deviations
TME: Tumor microenvironment
NHL: Non-Hodgkin’s lymphoma
