# Supplementary material for: CD74 as a prognostic and M1 macrophage infiltration marker in a comprehensive pan-cancer analysis
Source: Sci Rep. 2024 Apr 7;14:8125. doi: 10.1038/s41598-024-58899-7 (PMC10998849; doi:10.1038/s41598-024-58899-7)
Supplement: Supplementary file 1 — Supplementary Figures. [file 41598_2024_58899_MOESM1_ESM.docx]

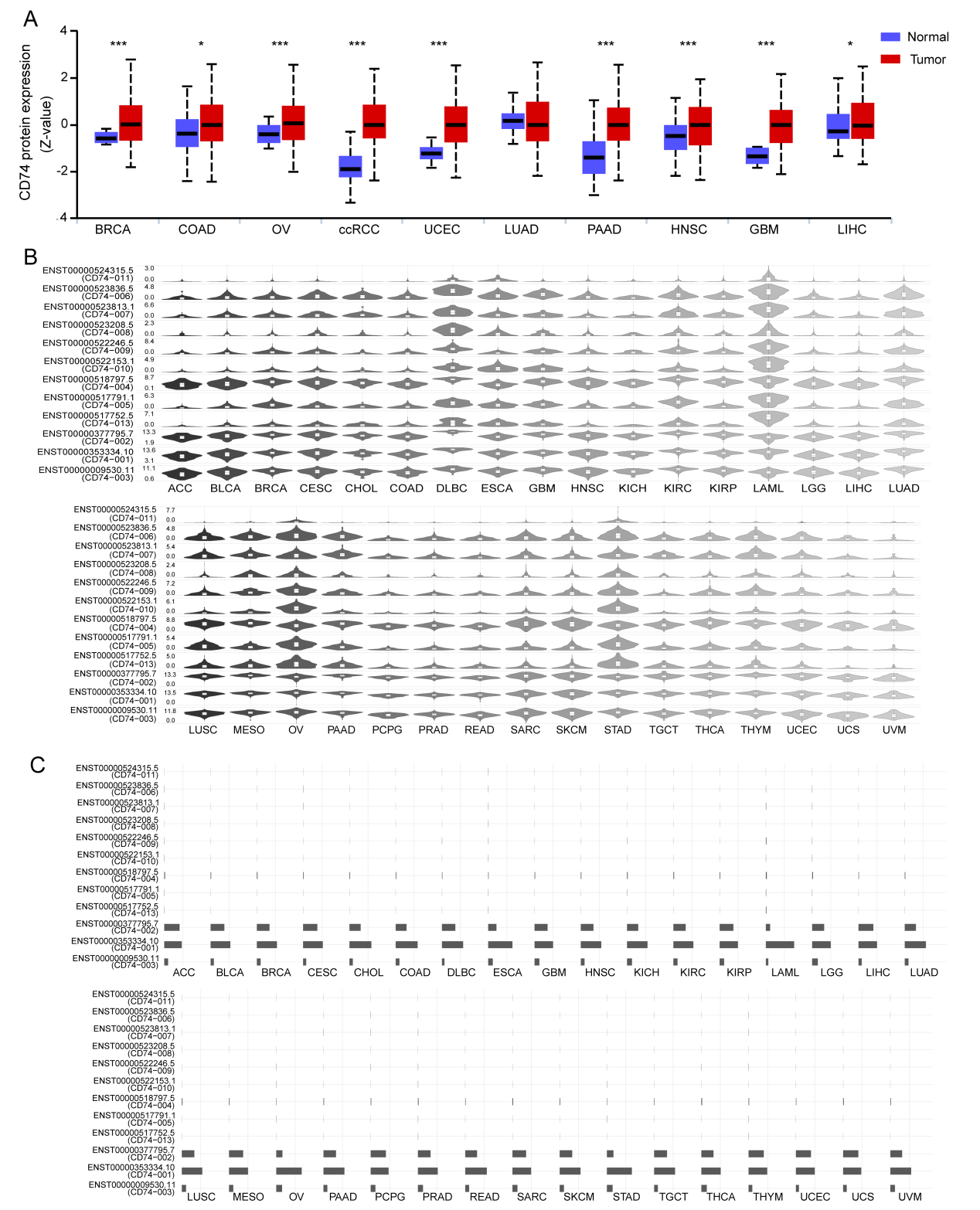


**Figure S1. CD74 protein levels and CD74 isoforms in pan-cancer.** (A) Comparison of protein levels between tumor and normal tissues, shown by UALCAN. (B) The violin-plots show the expression level (log2(TPM + 1)) of each isoform in CD74. (C) The bar-plot panel present the CD74 isoforms usage (from 0% to 100%) distribution.

**
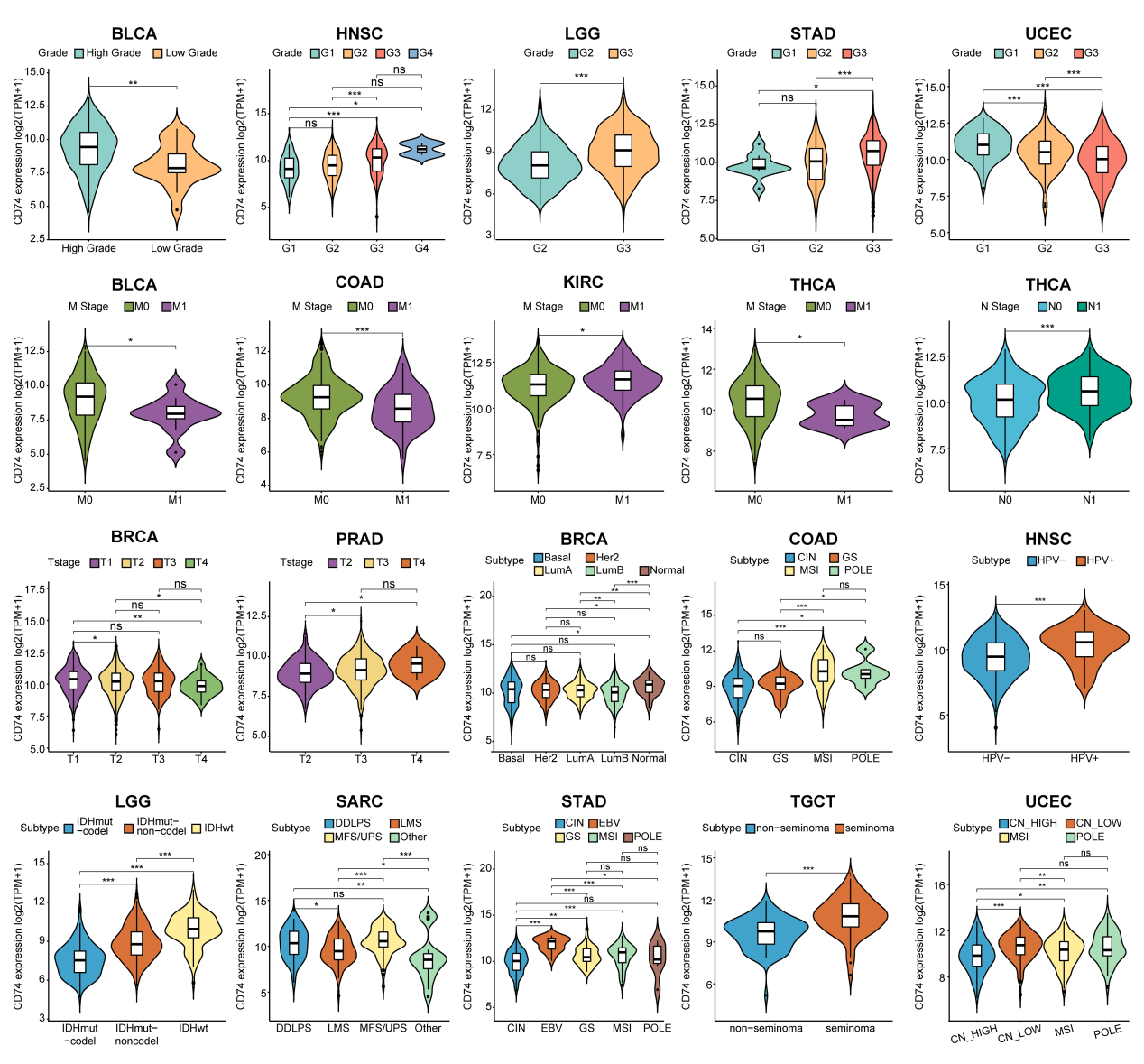
**

**Figure S2. CD74 mRNA levels in relation to tumor grade, TNM stage, and cancer subtype, from TCGA. ns, p≥0.05, *p<0.05, **p<0.01, ***p<0.001.**


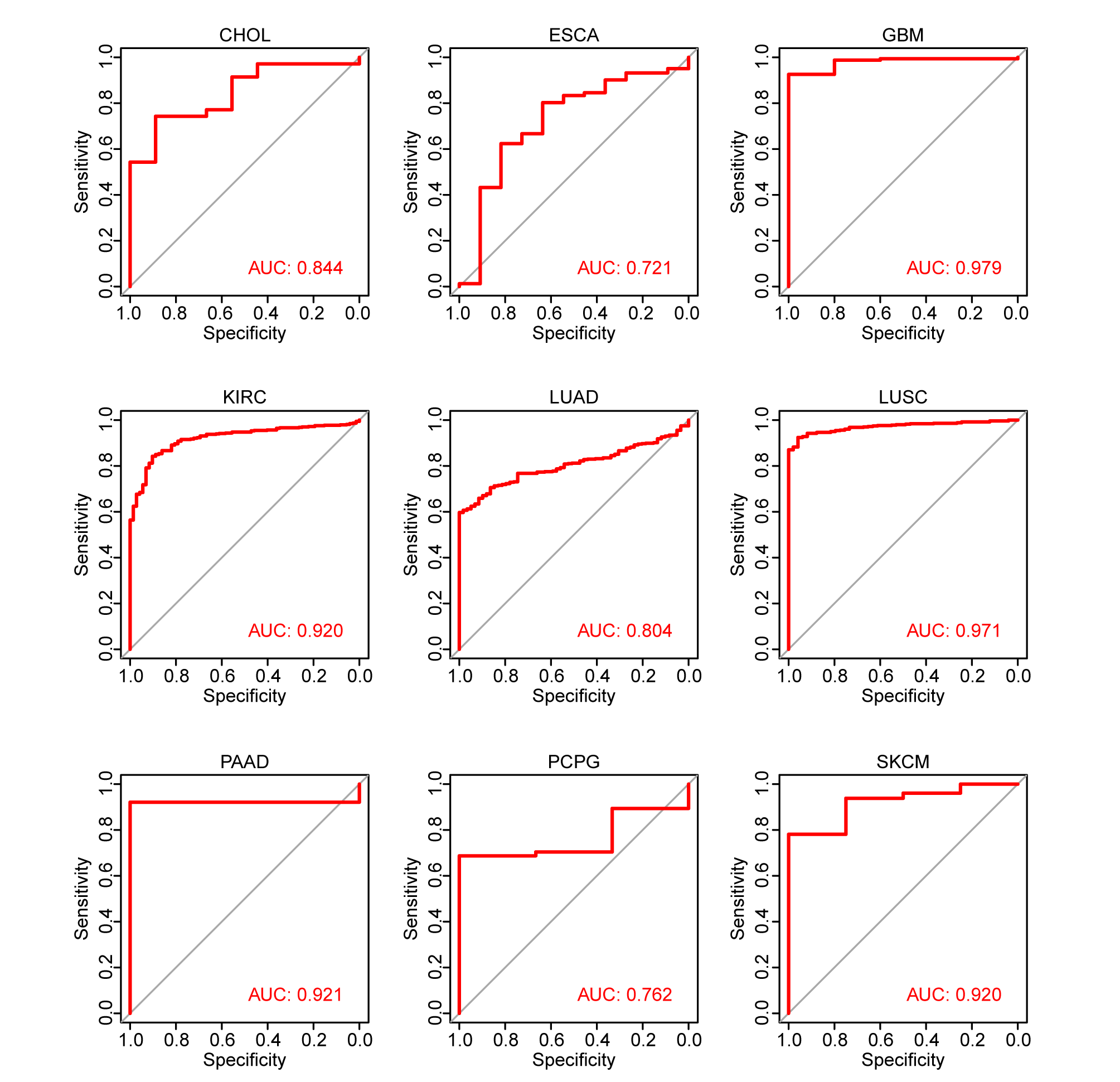


**Figure S3. The diagnostic ROC curves in pan-cancer.**


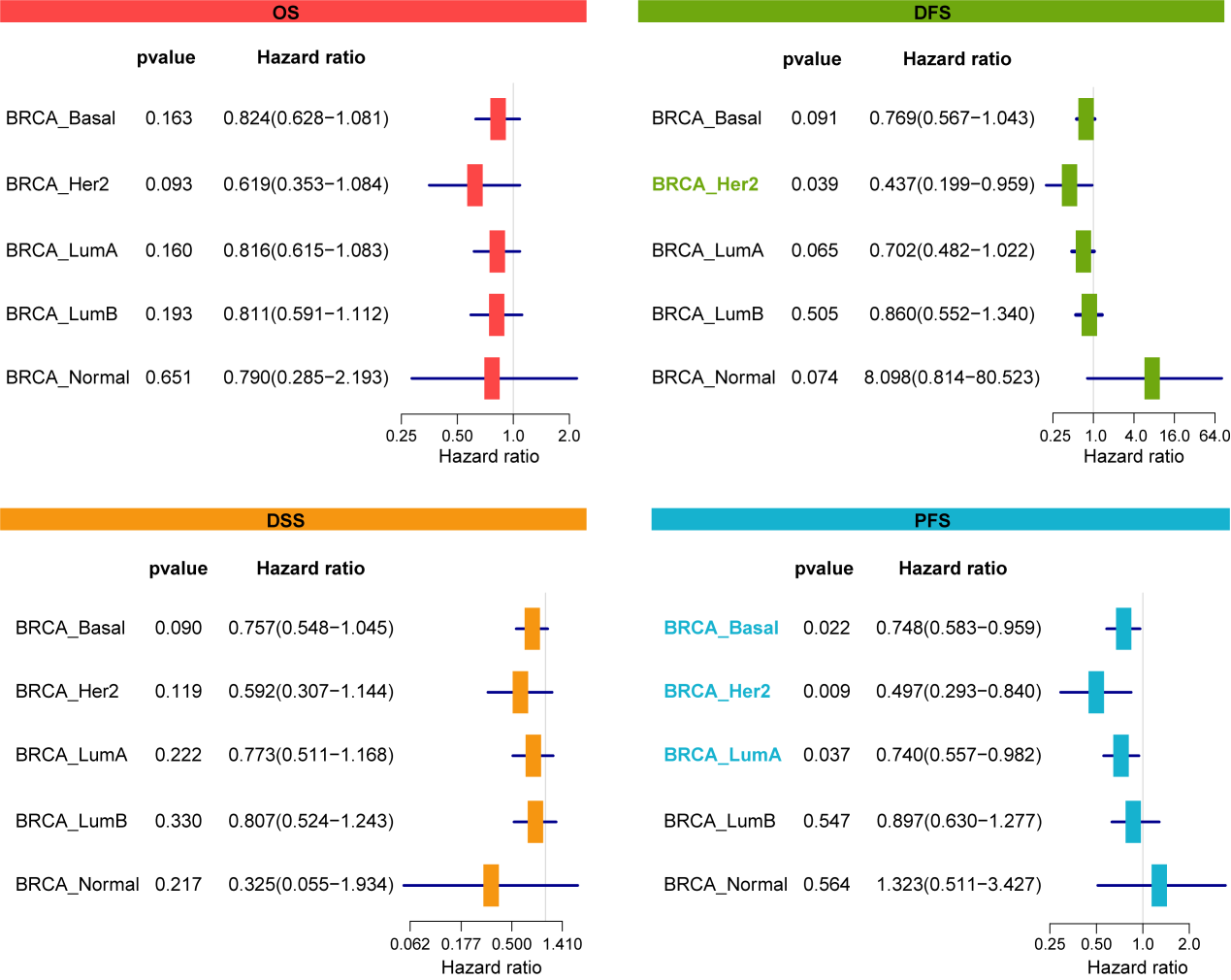


**Figure S4. The univariate Cox regression analysis for the association of clinical prognosis and CD74 in breast cancer subtypes.**


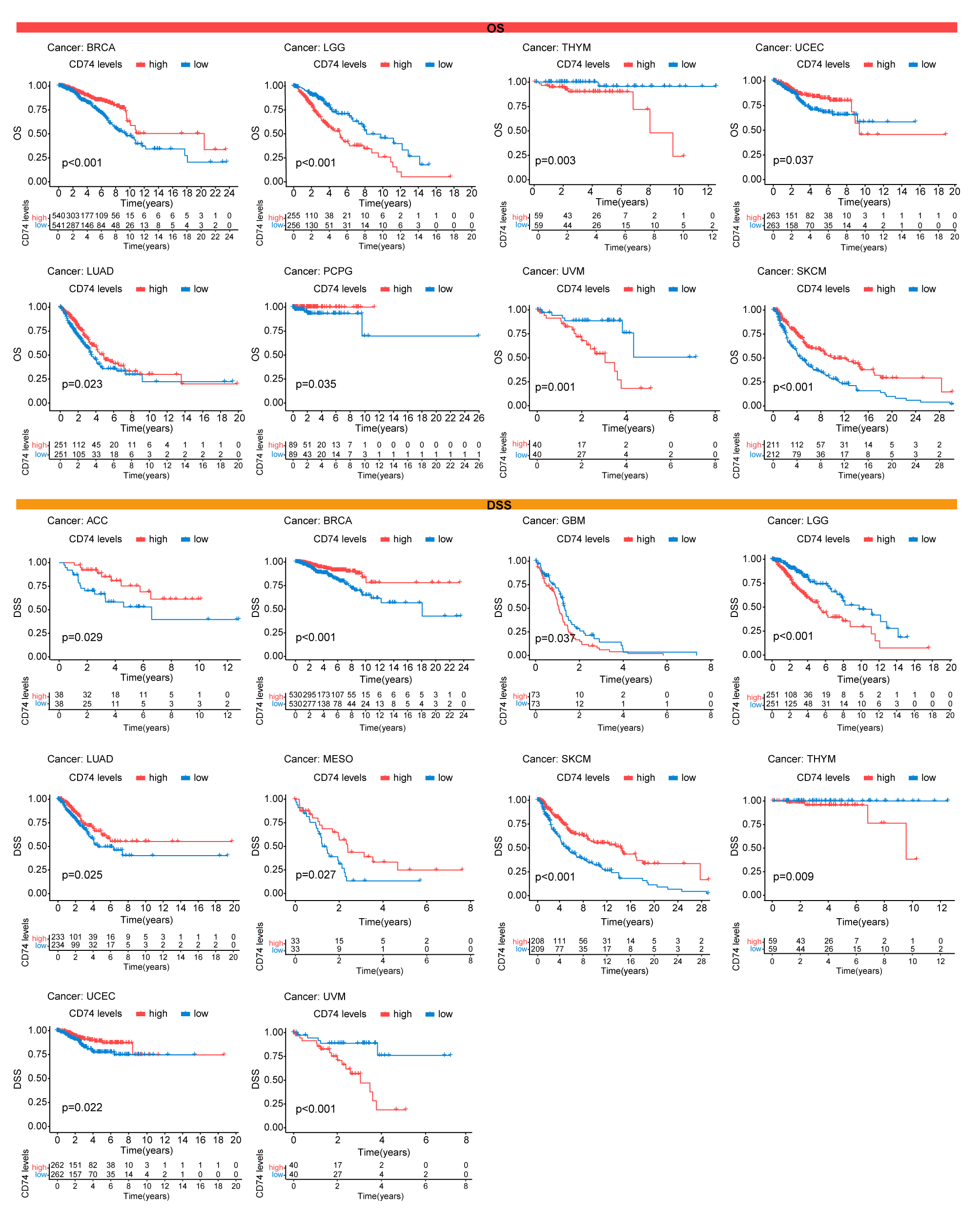


**Figure S5. KM curves showing associations between CD74 levels and OS and DSS in various cancers.**


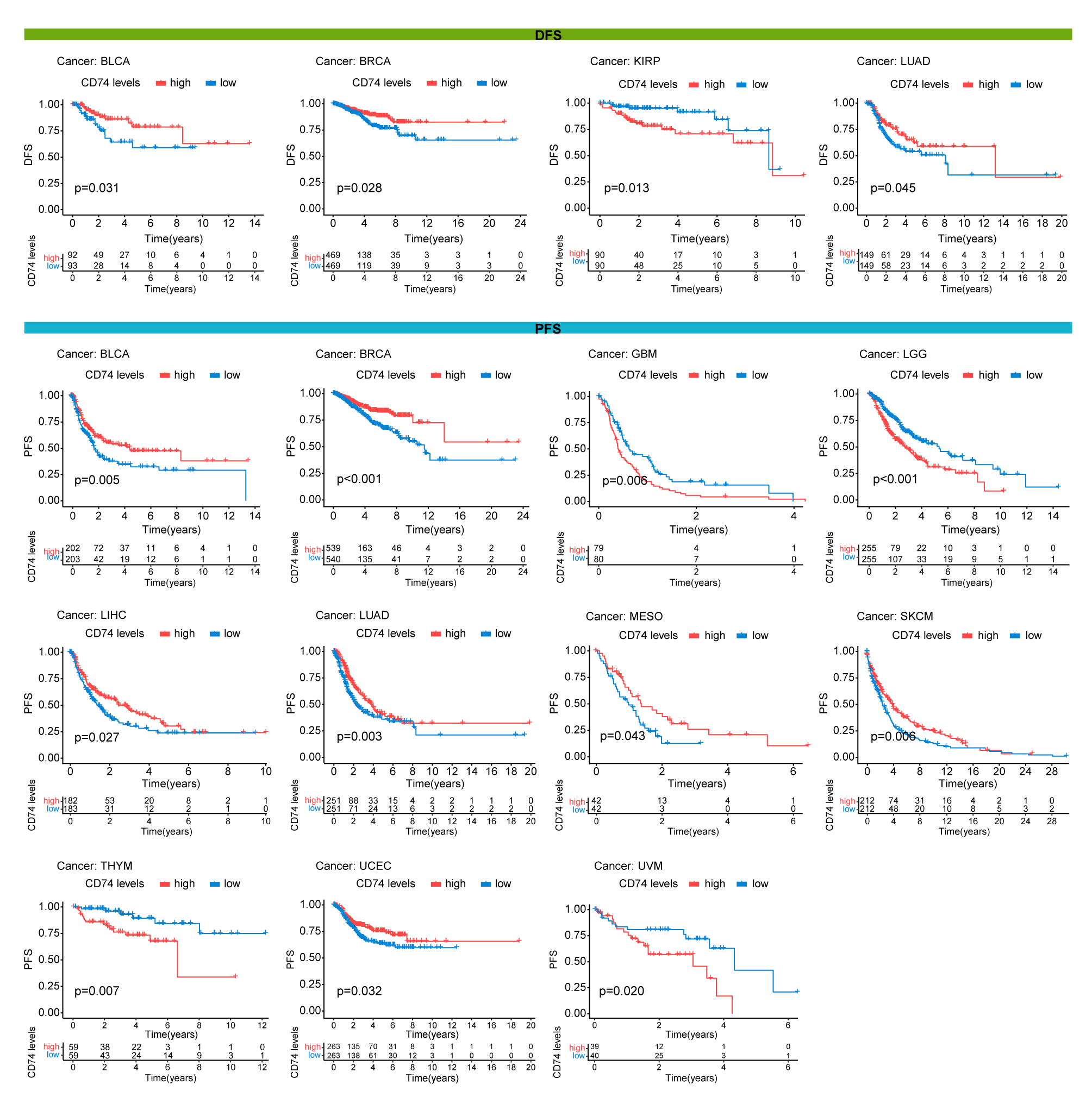


**Figure S6. KM curves showing associations between CD74 levels and DFS and PFS in various cancers.**


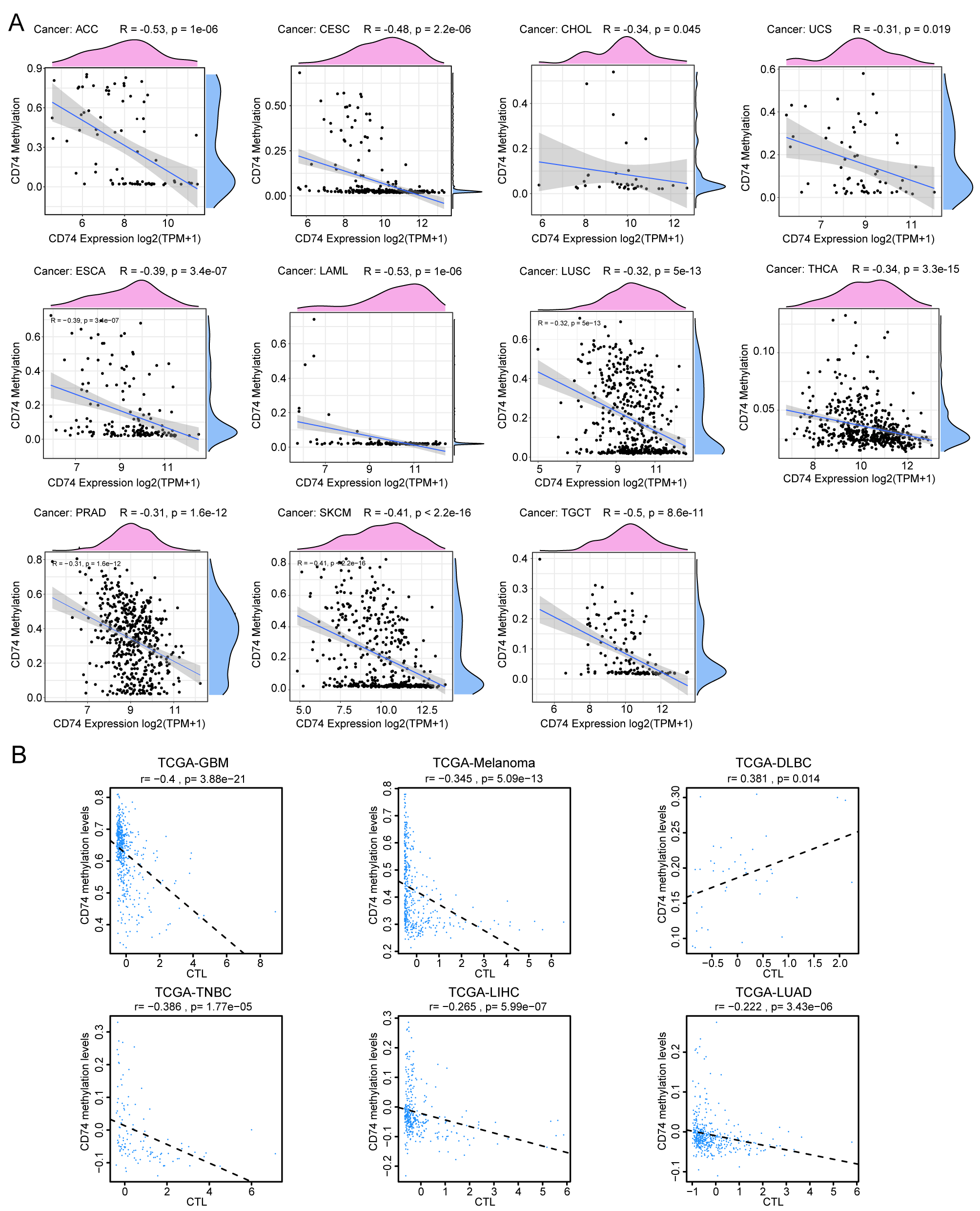


**Figure S7. Characterization of CD74 promoter methylation.** (A) Relationship between CD74 levels and promoter methylation in pan-cancer. (B) Scatterplots showing relationships between CD74 methylation and CTL markers.


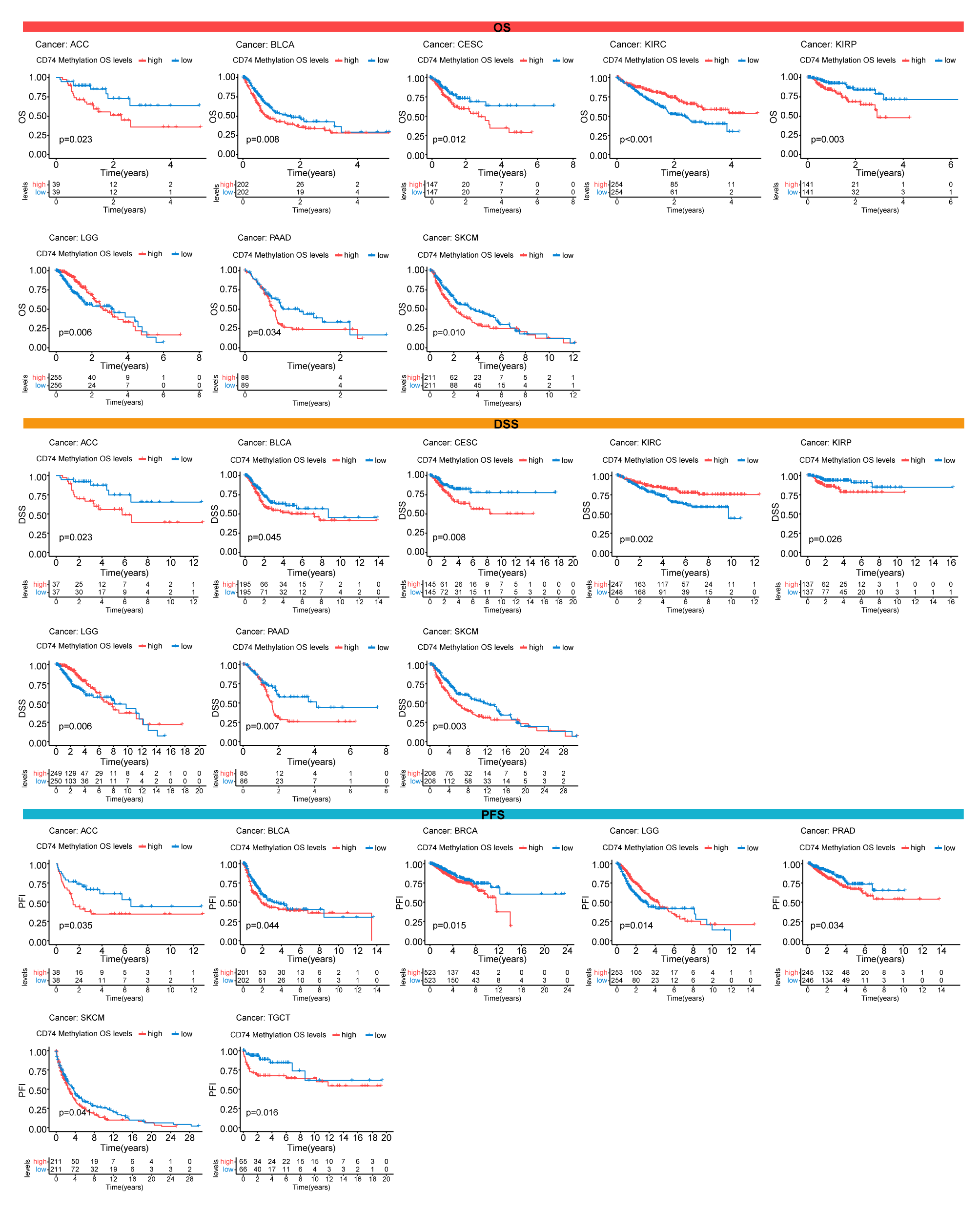


**Figure S8. Correlation of CD74 methylation levels with survival prognosis (OS, DSS, DFS, and PFS) in the distinct malignancies studied.**

**
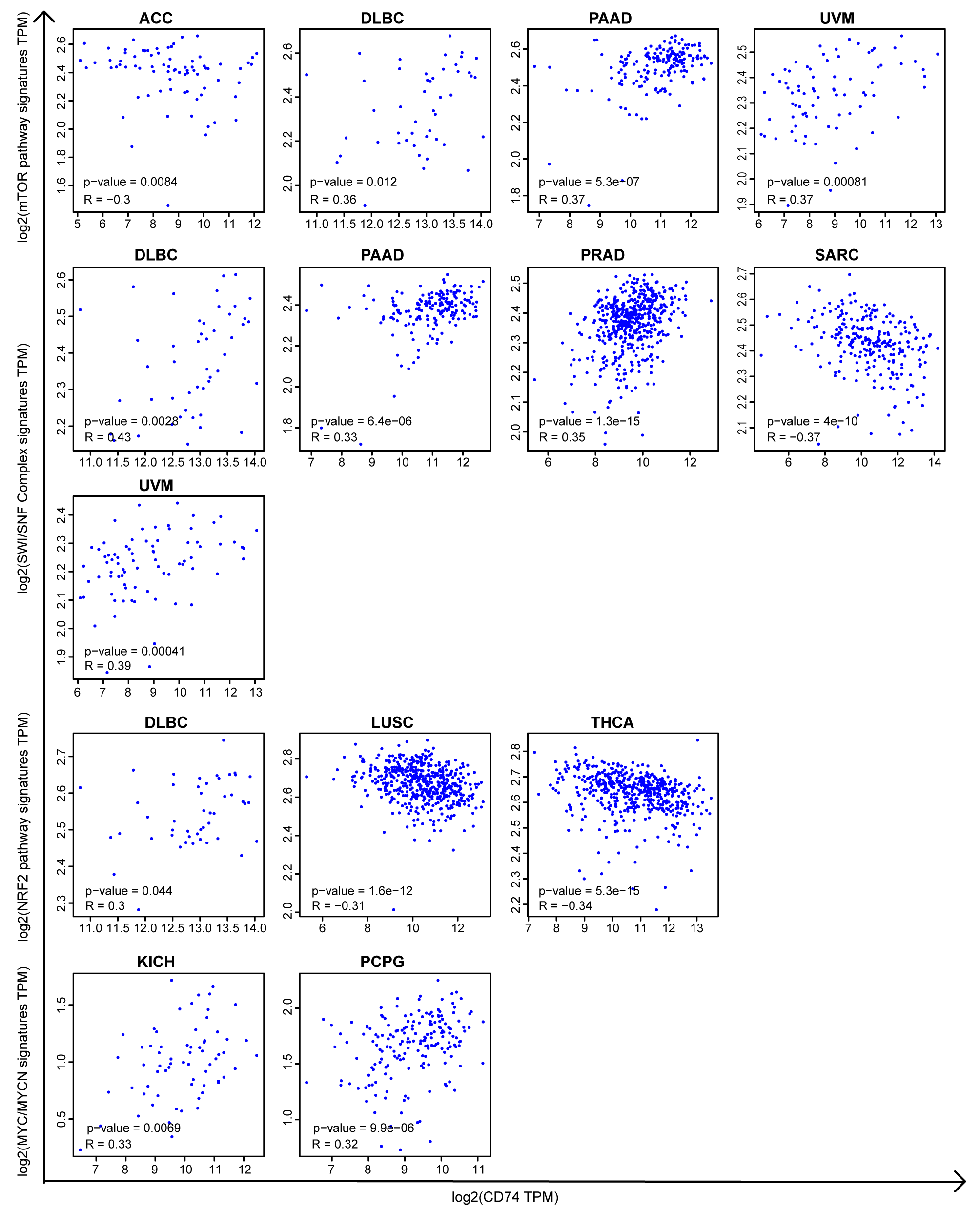
**

**Figure S9. Expression correlations between CD74 and oncogenic signaling pathways signature.**

**
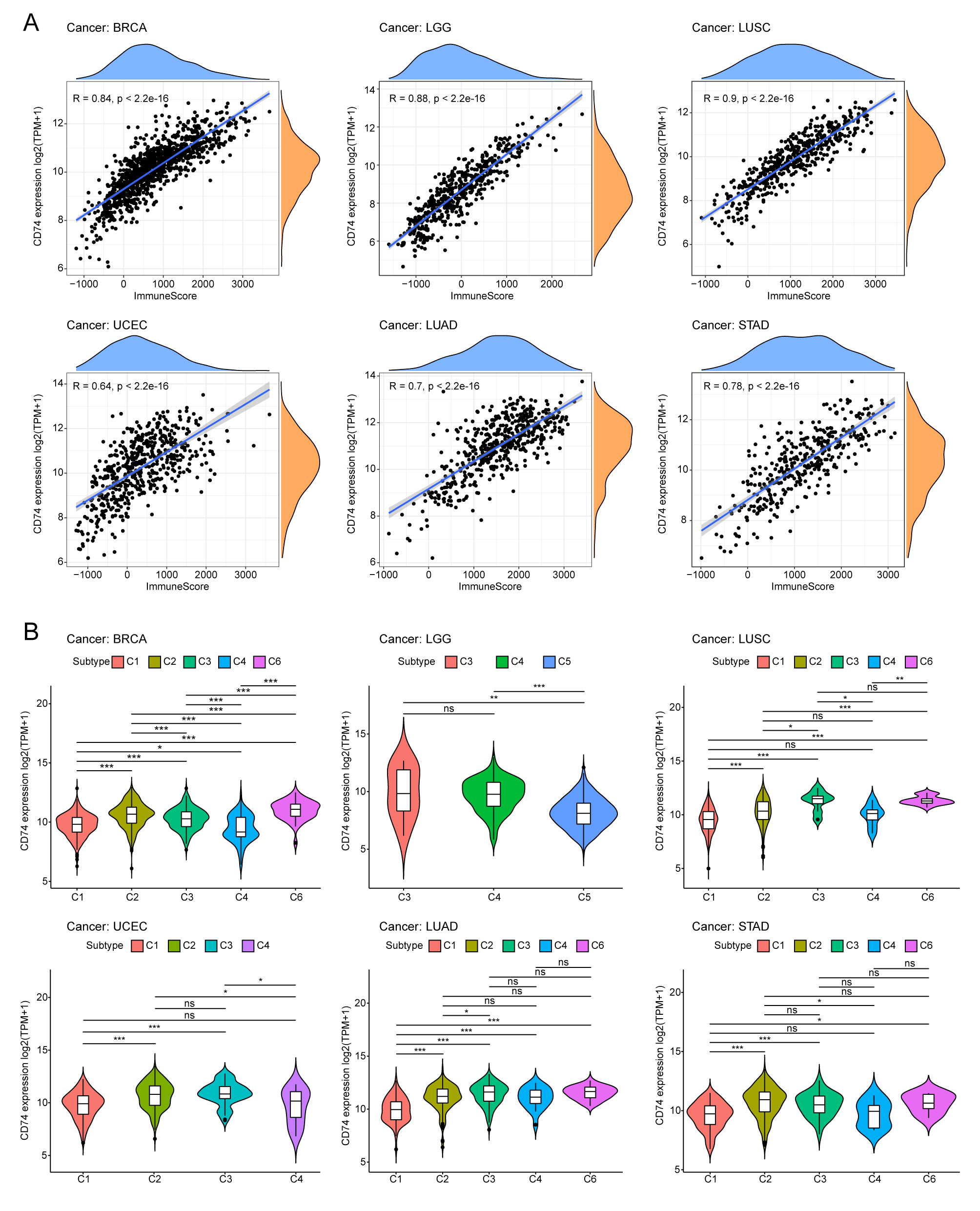
**

**Figure S10. The role of CD74 in tumor immunity.** (A) Scatterplots showing top six cancers in which CD74 levels are most closely associated with ImmuneScore. (B) CD74 levels in immune subtypes in BRCA, LGG, LUSC, LUAD, UCEC, and STAD. ns, p≥0.05, *p<0.05, **p<0.01, ***p<0.001.

**
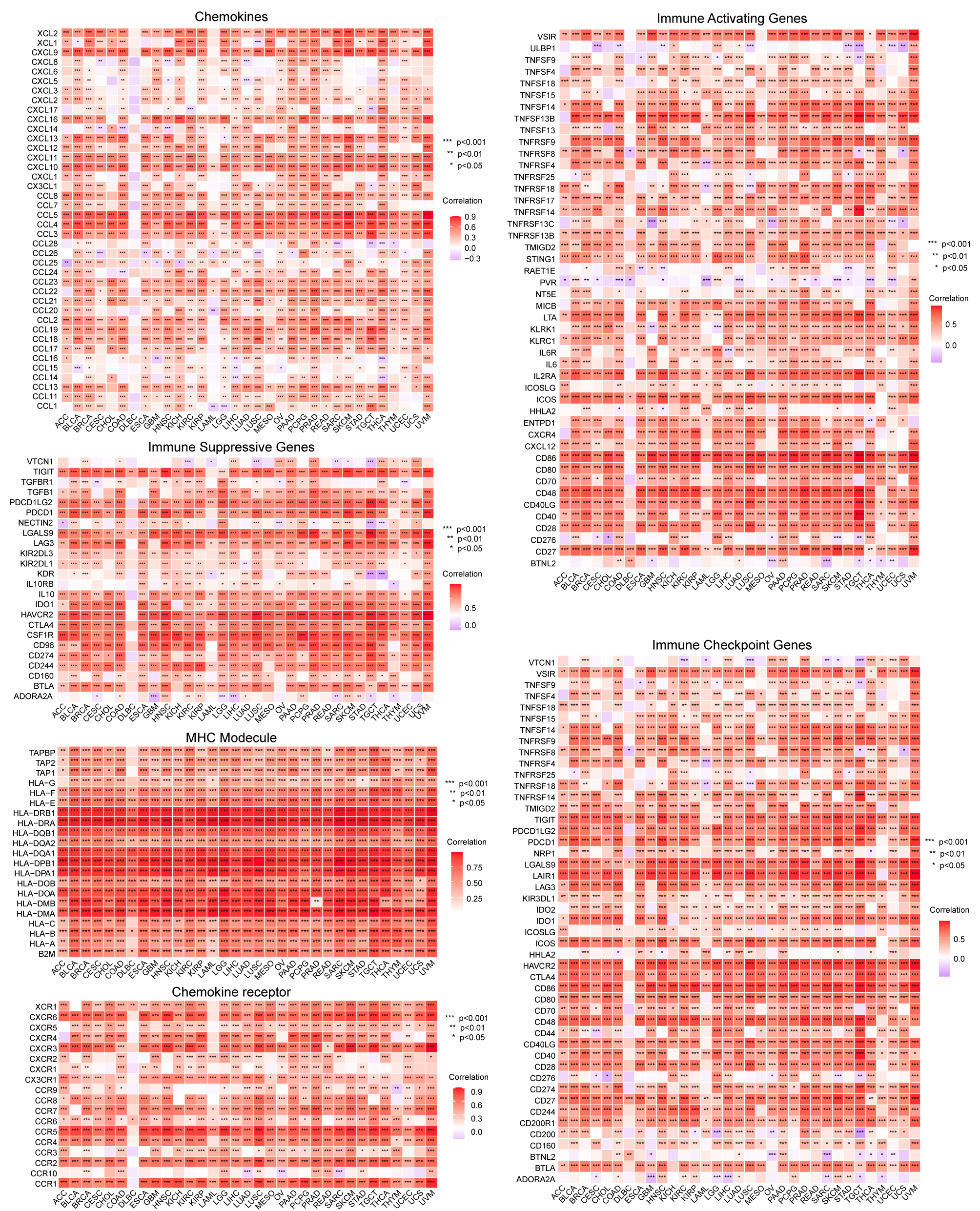
**

**Figure S11. Correlation between CD74 expression and immune-associated genes.** *p<0.05, **p<0.01, ***p<0.001.

**
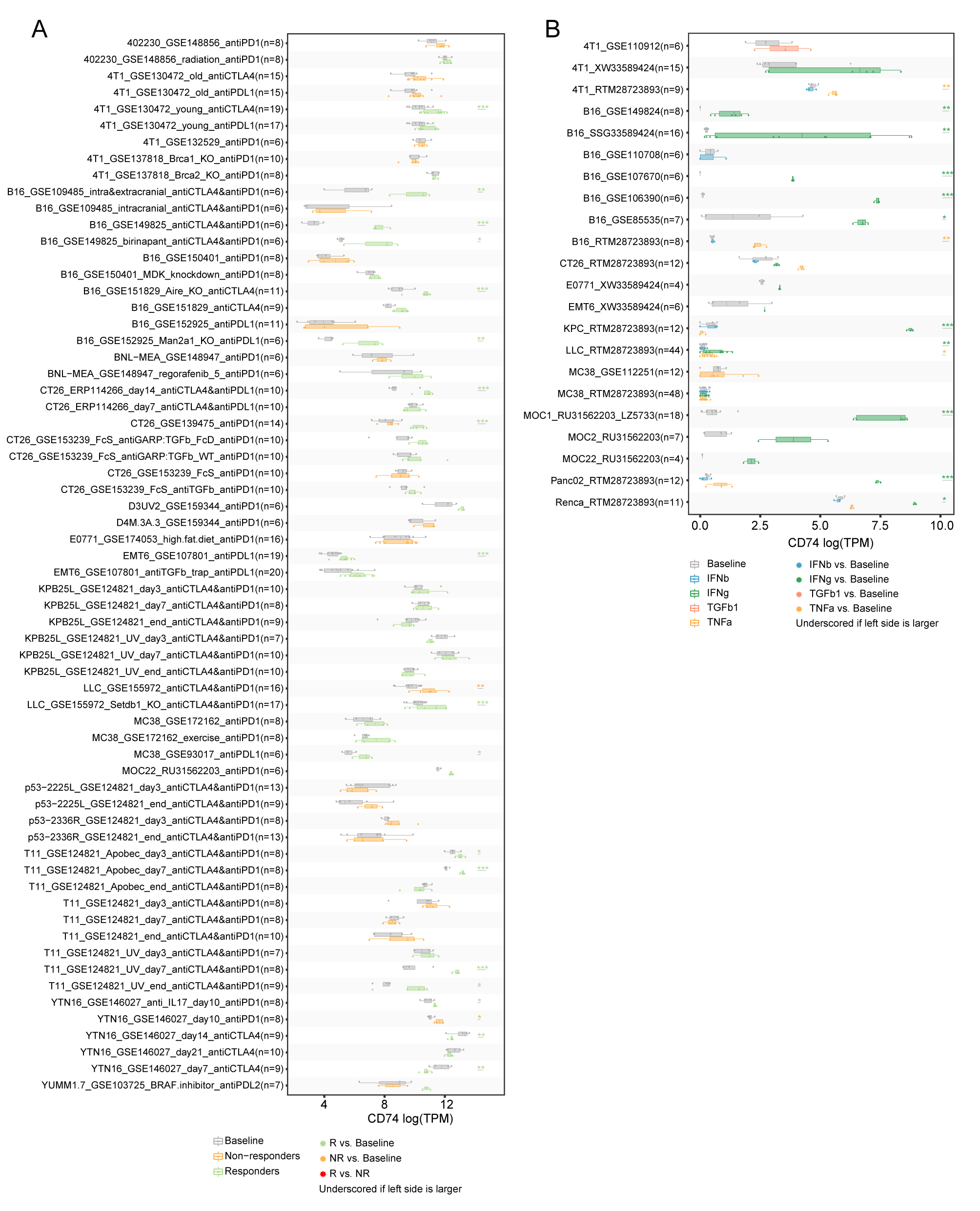
**

**Figure S12. CD74 levels before and after immunotherapy. (A)** Boxplots of CD74 levels before and after cytokine treatment, from TISMO. (B) Boxplots of CD74 levels before and after treatment with ICIs (anti-PD1, anti-PDL1, and anti-CTLA4), from TISMO. *p<0.05, **p<0.01, ***p<0.001.


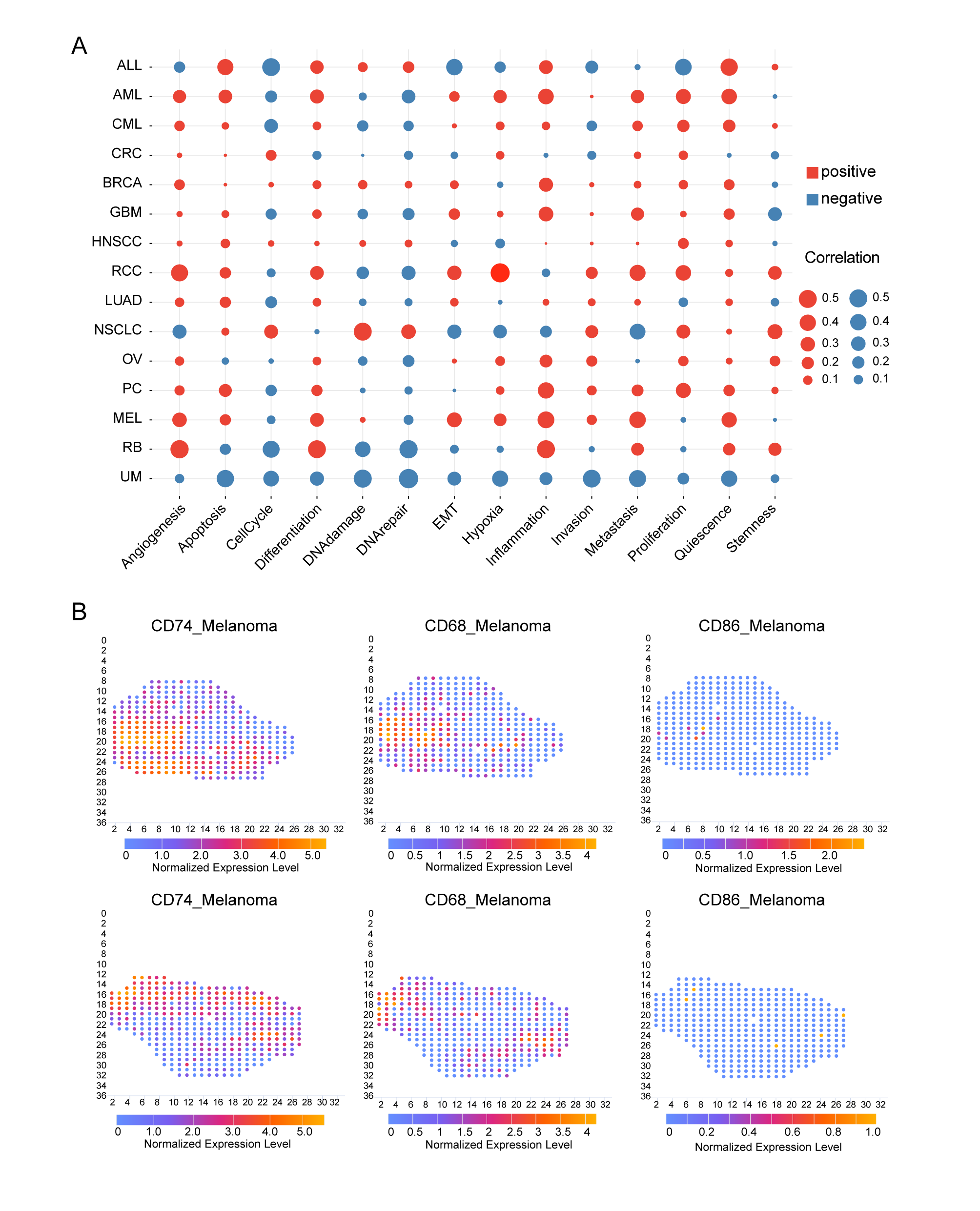


**Figure S13.** (A) Associations between CD74 and 14 functional cancer states, from single-cell sequencing data from CancerSEA. (B) Sections showing spatial distributions of CD74, CD68, and CD86 expression in melanoma. Colors of dots indicate the level of expression.
