# Supplementary material for: CD74 as a prognostic and M1 macrophage infiltration marker in a comprehensive pan-cancer analysis
Source: Sci Rep. 2024 Apr 7;14:8125. doi: 10.1038/s41598-024-58899-7 (PMC10998849; doi:10.1038/s41598-024-58899-7)
Supplement: Supplementary file 2 — Supplementary Information. [file 41598_2024_58899_MOESM2_ESM.docx]

**Supplementary Material S1 The FASTA sequence used for CD74 protein homology modeling.**

>NP_001020330.1 HLA class II histocompatibility antigen gamma chain isoform a [Homo sapiens]

MHRRRSRSCREDQKPVMDDQRDLISNNEQLPMLGRRPGAPESKCSRGALYTGFSILVTLLLAGQATTAYFLYQQQGRLDKLTVTSQNLQLENLRMKLPKPPKPVSKMRMATPLLMQALPMGALPQGPMQNATKYGNMTEDHVMHLLQNADPLKVYPPLKGSFPENLRHLKNTMETIDWKVFESWMHHWLLFEMSRHSLEQKPTDAPPKVLTKCQEEVSHIPAVHPGSFRPKCDENGNYLPLQCYGSIGYCWCVFPNGTEVPNTRSRGHHNCSESLELEDPSSGLGVTKQDLGPVPM
